# Extension of the Composite Quality Score (CQS) as an appraisal tool for prospective, controlled clinical therapy trials–A systematic review of meta-epidemiological evidence

**DOI:** 10.1371/journal.pone.0279645

**Published:** 2022-12-30

**Authors:** Steffen Mickenautsch, Stefan Rupf, Ivana Miletić, Veerasamy Yengopal

**Affiliations:** 1 Faculty of Dentistry, University of the Western Cape, Tygerberg, Cape Town, South Africa; 2 Department of Community Dentistry, School of Oral Health Sciences, Faculty of Health Sciences, University of the Witwatersrand, Parktown, Johannesburg, South Africa; 3 Review Center for Health Science Research, Bedfordview, Johannesburg, South Africa; 4 Chair of Synoptic Dentistry, Saarland University, Homburg, Germany; 5 Department of Endodontics and Restorative Dentistry, School of Dental Medicine, University of Zagreb, Zagreb, Croatia; Universidade de Sorocaba, BRAZIL

## Abstract

**Aim:**

To conduct a survey of current meta-epidemiological studies to identify additional trial design characteristics that may be associated with significant over- or underestimation of the treatment effect and to use such identified characteristics as a basis for the formulation of new CQS appraisal criteria.

**Materials and methods:**

We retrieved eligible studies from two systematic reviews on this topic (latest search May 2015) and searched the databases PubMed and Embase for further studies from June 2015 –March 2022. All data were extracted by one author and verified by another. Sufficiently homogeneous estimates from single studies were pooled using random-effects meta-analysis. Trial design characteristics associated with statistically significant estimates from single datasets (which could not be pooled) and meta-analyses were used as a basis to formulate new or amend existing CQS criteria.

**Results:**

A total of 38 meta-epidemiological studies were identified. From these, seven trial design characteristics associated with statistically significant over- or underestimation of the true therapeutic effect were found.

**Conclusion:**

One new criterion concerning double-blinding was added to the CQS, and the original criteria for concealing the random allocation sequence and for minimum sample size were amended.

## Introduction

According to the Cochrane Collaboration, the risk of bias in controlled clinical therapy trials, more specifically randomised controlled trials (RCTs), should be appraised using its Risk of Bias (RoB) tool, Version 2 (RoB 2) [1]. The tool comprises five bias domains concerning the randomisation process, deviation from intended interventions, missing outcome data, measurement of outcome and selection of the reported result. Within each domain, a series of questions (’signalling questions’) aim to elicit information about features of the trial that are relevant to the risk of bias. A proposed judgement about the risk of bias arising from each domain is generated by an algorithm, based on answers to the signalling questions. Following such assessment, the overall bias risk of an RCT is labelled as: ’low’, ’some concern’ or ’high’. If, for an RCT, all five bias domains of the RoB 2 tool have been assessed as low bias risk, then the entire trial is judged as ‘low bias risk’ [1].

With the RoB tool, trials are appraised using a particular set of criteria, which, if fully complied with, form the basis for the judgement that the bias risk of the appraised trial is ‘low’. However, it is our contention that confidence in low bias risk cannot be justified, because only a limited number of trial characteristics were observed when using the RoB tool. Such confidence further relies on the assumption that any unobserved trial characteristic outside the applied set of appraisal criteria did not bias the trial results [2]. The correctness of such an assumption cannot be proven [3]. Instead, it has been recognised that any single systematic or random error can completely invalidate the results of the trial [4]. Such an error will remain undetected when it lies outside of the applied set of trial appraisal criteria. It follows that judging a trial by use of the RoB tool as of ‘low bias risk’ may be misleading.

In addition, trial evidence appraisal requires high inter-rater reliability in order to avoid the risk that the appraisal results are rater-dependent. Rater-dependent results have little utility in the judgement of whether trial results are valid, due to a high level of observer variability and thus a very high risk of measurement error [5]. To date, no information could yet be found for the RoB 2 tool according to the Landis/Koch Kappa’s Benchmark Scale [5]. However, studies considering the first version of the RoB tool have established only ‘poor’ to ‘moderate’ inter-rater reliability [6–9]. For these reasons, clinical trial appraisal tools with a more robust epistemic basis and higher inter-rater reliability are needed.

The Composite Quality Score (CQS) is a novel tool for the appraisal of prospective, controlled clinical therapy trials [9]. In its original form, the CQS consists of three simple appraisal criteria that are a composite of trial appraisal categories for both systematic and random error. The CQS does not rely on the assumption that any unobserved trial characteristic outside the applied set of appraisal criteria would not bias the trial results [10]. Consequently, no certainty of ‘low bias risk’ is ascribed to an overall positive CQS appraisal result, regardless of how many appraisal criteria any trial has complied with. Instead, the CQS follows the concept that, although ‘low bias risk’ cannot be proven, it is always possible to establish ‘high bias risk’ with high certainty [2]. ‘High bias risk’ is established when any trial characteristic that is essential for a true trial result is absent.

Mickenautsch et al. (2021) investigated the CQS inter-rater reliability. Their results showed an almost perfect inter-rater reliability for the CQS, according to the Landis/Koch Kappa’s Benchmark Scale [5]; that is, a very low level of observer variability and thus a very low risk of measurement error: Brennan-Prediger coefficient 0.95 (95% CI: 0.87–1.00). During the same study, the CQS reliability was directly compared to that of the first version of Cochrane’s RoB tool. The reliability of the latter ranged between –0.07 (95% CI: -0.42–0.28) and 0.34 (95% CI: -0.05–0.73) [9]. Most of the differences between the RoB and the CQS were statistically significant (p < 0.05).

The current number of three CQS criteria appears to have been sufficient for clinical trial appraisal in the field of restorative dentistry [11], where only two from the total of 683 trial reports were not rated as of low quality of evidence (QoE). However, other fields of clinical therapy may contain a higher number of trials that would pass the three simple and non-restrictive CQS criteria. Since such trials would be eligible for further appraisal, the CQS would need to be extended by new criteria in order to be useful. Such CQS extension is open-ended and may commence as long as new criteria can be justified.

Systematic or random errors may divert established effect estimates in trials from the true therapeutic intervention effect in the form of either an over- or underestimation of such effect. The risk of such error is related to the characteristics of the applied trial method. Trial appraisal criteria aim to establish whether such characteristics are present in a trial or not. However, such criteria themselves will need to be based on sound reasons [10]. It is considered that sound reasons are given when empirical evidence from meta-epidemiological studies shows that the absence of a particular trial design characteristic is associated with a statistically significant (p < 0.05) effect of over- or underestimation. Meta-epidemiological studies are ‘studies on studies’ that compare intervention effect estimates between clinical trials with or without a particular trial design characteristic [12]. Ideally, the results of meta-epidemiological studies provide the basis for inferring how the lack of a characteristic, for instance the lack of randomised allocation sequence generation or lack of patient blinding, affects the results of clinical trials in terms of either an over- or underestimation of the true therapeutic intervention effect.

For this reason, the aim of this study was to conduct a survey of current meta-epidemiological studies, based on a systematic literature search, to identify additional trial design characteristics that may be associated with a significant over- or underestimation of the treatment effect and to use such identified trial design characteristics as a basis for the formulation of new CQS appraisal criteria.

## Materials and methods

### Survey of current meta-epidemiological study evidence

The methodology of this study was pre-specified in a protocol, which was made available online prior to the start of the study [13]. As far as applicable, this study is reported according to the PRISMA statement [14] (see S1 File).

#### Eligibility criteria

We included any meta-epidemiological study that fulfilled the following criteria:

- Investigation of the association between any specific trial design characteristics and intervention effect estimates in prospective, clinical controlled therapy trials;
- Adoption of a matched design in the study’s methodology (matching of trials with similar clinical scenarios, including the type of studied populations and interventions, selected comparators and measured outcomes);
- Inclusion of computation concerning the effect of a specific trial design characteristic on the intervention effect estimate;
- Reporting of the computed effect estimate, for instance as Ratio of Odds Ratios (ROR), Ratio of Hazard Ratios (RHR) or any other form of effect estimates, for binary estimates or the Difference in Standardised Mean Differences (dSMD) for continuous estimates, together with the associated in-between meta-analysis or in-between trial heterogeneity.

In the case of two or more meta-epidemiological studies including the same meta-analyses or trials for the same trial design characteristic, the studies with the least in-between meta-analysis (or trial) heterogeneity were selected.

Studies were not included if:

- No clearly computed effect estimate related to a trial design characteristic could be extracted;
- The included comparison(s) relates to trial differences outside prospective, clinical controlled study design characteristics.

#### Search strategy

We included the systematic review by Page et al. (2016) [15] for direct data extraction and, together with the systematic review by Dechartres et al. (2016) [16], searched the reference lists for additional suitable studies.

In the first review, Page et al. (2016) identified a total of 3081 records and retrieved 118 full-text articles, published up to May 2015. We retrieved all meta-epidemiological studies that had been initially selected. We also included studies for all comparisons of effect estimates with trial characteristics that were deemed ineligible by the review authors. In the second systematic review, Dechartres et al. (2016) identified a total of 941 citations from which 56 meta-epidemiological studies, published up to April 2015, were initially selected.

To identify more recent studies, we searched PubMed (June 2015 to March 2022) using the search term ‘meta-epidemiolog*’, and Embase (June 2015 to March 2022) using the search term ‘meta-meta-anal$ OR meta-review$ OR meta-epidemiologic$ OR metaepidemiologic$’ (all fields + text). In addition, we also searched the reference lists of all studies that were identified during the database search.

#### Study selection

One reviewer (SM) conducted the searches by screening citation titles and abstracts and retrieved the full-text articles. A second reviewer (SR) independently verified the retrieved articles for eligibility. Any disagreements were resolved via discussion and consensus.

#### Data extraction and management

One reviewer (SM) extracted and entered all data into a Microsoft Excel sheet. SR verified the data entry for accuracy. The following data were extracted:

- Comparison type(s) between trial design characteristic(s) per meta-epidemiological study;
- Number of meta-analyses and/or trials per comparison type included in the study;
- Reported effect estimate type and values per comparison type, for example ROR, RHR for binary estimates or Difference in Standardised Mean Differences (dSMD) for continuous estimates, or whichever is reported by the study investigators;
- Reported type and values of measure for estimate precision per comparison type, for example Confidence Interval (CI) or Credible Interval (CrI);
- Reported type and values of in-between meta-analysis or trial heterogeneity measure (e.g. I^2^, τ^2^, φ); and
- Direction of effect for overestimation.

#### Statistical analyses

We statistically pooled effect estimates and measures of precision from several meta-epidemiological studies per trial design characteristic. Pooling was not conducted if:

- Studies included similar meta-analyses/trials with regard to the same comparisons types;
- Definitions of trial design characteristics were non-comparable between studies;
- Types of reported effect estimates, types of precision measures or the direction of the effect differed;
- Numbers of meta-analyses/trials per comparison were not reported or where only the number of meta-analyses was reported in one and only the number of trials reported in the other.

Since the included studies may have varied in their trial design characteristics, we used random-effects meta-analysis for pooling. DerSimonian and Laird’s method of moments estimator was used to estimate the between-study variance [17]. Statistical inconsistency was quantified using the I^2^ statistic [16]. To pool effect estimates for binary and continuous outcomes, dSMD values were converted to log RORs by multiplying by π/√ 3 = 1.814 [18].

### Formulation of new CQS appraisal criteria

Only trial design characteristics for which a statistically significant (p < 0.05) effect over- or underestimation could be established were taken as a basis for the formulation of new CQS appraisal criteria, provided these were amenable to established CQS principles [10]:

- The criteria needed to allow high confidence of low QoE based on a negative (No = 0) appraisal result and thus would have needed to be least stringent.
- Where CQS criteria for certain error domains already existed, the new criteria required incrementally, minimally higher stringency than the existing ones.

Each CQS criterion is used to appraise trials for a specific error and was designed sufficiently lenient. Therefore, the presence of an error can be expected with high confidence in studies that fail to meet any such criterion. Trial design characteristics were not considered for CQS extension if:

1. It provided low confidence for low QoE, for example any characteristic that is based on a comparison between high and low bias risk as defined by Cochrane’s RoB tool (since any definition of ‘low bias risk’ cannot be justified);
2. No statistically significant effect estimate has been established in meta-epidemiological studies;
3. Where more than one dataset with contradicting effect magnitudes (statistically non-significant, as well as significant) are found but which cannot be pooled.

In future updates, the CQS may be further extended or amended based on new meta-epidemiological evidence that could not be identified within the scope of this study.

## Results

### Search results and data extraction

**Fig 1:**
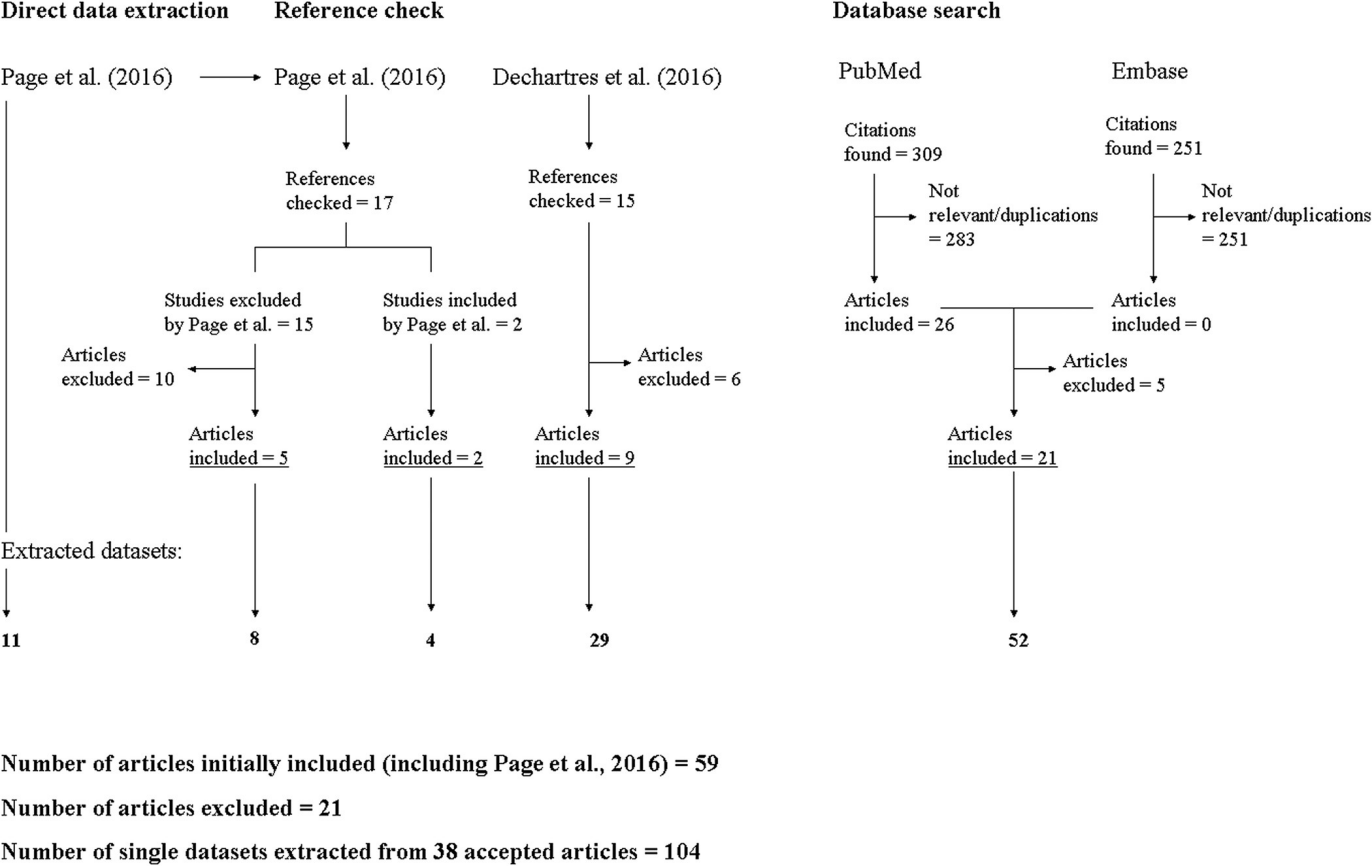
Flow diagram of identification, screening and inclusion of articles and datasets.

Our own database search from June 2015 –March 2022 identified a total of 560 citations. From the reference check of both systematic reviews [15, 16] and from our own database search, a total of 33 (including the article by Page et al. [15]) and 26 meta-epidemiological studies were included respectively (Fig 1). From these, 21 studies were excluded (see S2 File Page 2) and 38 studies accepted (see S2 File Page 3 and S3 File).

Reasons for study exclusion were: (i) No computed comparison result related to a trial design characteristic extractable (14 studies); (ii) Comparison related to differences outside prospective, clinical controlled study design characteristics (1 study); (iii) Lack of relevance (4 studies); (iv) Older version of included study (1 study), and (v) Duplication (1 study).

From the 38 accepted studies, a total of 104 single datasets were extracted (Fig 1) and are listed in S2 File Page 6. From these, 76 datasets were excluded for the following reasons: (i) Low confidence for low QoE (N = 5); (ii) Datasets with statistically significant results for a trial design characteristic for which datasets with non-significant results were also found but which cannot be pooled (N = 15); (iii) Not relevant for prospective, clinical controlled trials (N = 2); (iv) No statistically significant effect estimate established (N = 53); (v) Duplication of reported data (N = 1). A list of the excluded datasets is provided in S2 File Page 10.

A total of 21 datasets were identified as sufficiently homogeneous for possible pooling in 10 separate meta-analyses. Details of these are presented in S2 File Page 14. Two groups of datasets were excluded; one (MA-ID 5) due to lack of relevance for prospective, clinical controlled trials, and the other (MA-ID 8) due to its trial design characteristics providing only low confidence for low QoE.

In addition to the eight groups of datasets, accepted for meta-analysis, a further six single datasets that were unsuitable for meta-analysis but yielded statistically significant results were included as a basis for possible CQS extension (S2 File Page 18).

### Meta-analysis and single datasets for CQS extension

The results of all eight meta-analyses are presented in S2 File Page 15. Four of the eight meta-analyses showed statistically non-significant results and were excluded from CQS extension. The results of the meta-analyses and single datasets that were included for CQS extension are presented in Table 1. The trial design characteristics for which statistically (p < 0.05) significant effect estimates could be established were: allocation concealment; double-blinding; intention-to-treat analysis; multi-centre study design (for continuous outcomes) and sample size limit (>100; >500 and >1000 patients).

**Table 1:** Meta-analysis and single dataset results included for CQS extension.

Lack of trial design characteristic	Meta-analysis result			Single dataset result				
	Effect estimate	N	ID	Effect estimate	N	ID	Study ID	Study reference
Inadequate or unclear allocation concealment	dSMD 0.15; 95% CI 0.03 to 0.28; I^2^ = 0%	379	02	-	-	-	-	-
No or unclear double- blinding	-	-	-	ROR 0.91; 95% CI 0.84 to 0.99	467	36	47	Martin et al. (2021) [19]
				ROR 0.87; 95% CrI 0.79 to 0.96	1057	103	74	Savović et al. (2012) [20]
Deviation from standard intention-to-treat analysis	-	-	-	ROR 0.80; 95% CI 0.69 to 0.94	202	8	11	Abraha et al. (2015) [21]
Multi-centre study design for continuous outcomes	ROR 0.78; 95% CI 0.68 to 0.91; I^2^ = 56.6%	713	04	-	-	-	-	-
Sample size limit <100	ROR 0.67; 95% CI 0.54 to 0.82; I^2^ = 80.2%	919	09	ES –0.21; 95% CI –0.34 to– 0.08	153	81	73	Nüesch et al. (2010) [22]
Sample size limit <500	-	-	-	ROR 0.81; 95% CI 0.74 to 0.89	434	79	72	Dechartres et al. (2013) [23]
Sample size limit <1000	-	-	-	ROR 0.82; 95% CI 0.76 to 0.90	301	80	72	

N = Number of trials included: CI = Confidence interval; CrI = Credibility interval; ROR = Ratio of Odds Ratios; ES = Effect size; dSMD = Differences in Standardised Mean Differences.

### Formulation of new CQS appraisal criteria

#### Double-blinding

The results of two studies, by Martin et al. (2021) [19] and Savović et al. (2012) [20], indicated a statistically significant larger effect estimate for trials in which double-blinding was not clearly applied (either RCTs where it was explicit in the methodology that there was no double-blinding or where it was unclear whether double-blinding was done), with an overestimation of 9% (ROR 0.91; 95% CI: 0.84–0.99) and 13% (ROR 0.87, 95%CrI: 0.79–0.96), respectively. The evidence from both studies combined the results of 1525 double-blinded and non-double-blinded clinical trials in the fields of pregnancy and childbirth circulatory system conditions, mental health [20] and pharmacological interventions that covered a wide range of critical care pathologies [19]. In the former study, the term ‘double-blind’ was defined as an intervention that has been unknown to patients and personnel and the term ‘non-double-blind’ was related to the descriptions: ‘single-blind’, ‘open-label’ and ‘distinguishable intervention’, as well as ‘unclear’ or when no information was given. In the latter study, the definitions varied, including any two of the three distinctive groups: trial participants, trial personnel and trial outcome assessors being blinded to the applied interventions. Nonetheless, the inter-study reliability in the assessment of this trial design characteristic was high (80–100% agreement in 4 of 9 comparisons, with κ - statistics ranging from 0.55–1.00, median 0.87) [20]. Subgroup analysis showed a higher overestimation effect in non-double-blinded trials with subjective outcomes than with objective ones [20]. However, the study by Martin et al. (2021) [19] indicated statistically significant higher effect estimates in non-double-blinded trials with the objective outcome ‘mortality’ (long- and short-term combined), too, suggesting that effect overestimation is not limited to non-double-blinded trials with subjective outcomes, only.

Based on the established evidence the following CQS criterion was formulated:

### Double-blinding or the blinding of at least two out of the three groups: trial participants; trial personnel and trial outcome assessors in some form reported in the text (Yes = 1 / No = 0)

In this context, trial participants are considered as patients receiving treatment in the trial intervention groups; trial personnel are the clinical staff that administer such treatment and trial outcomes assessors are those who collect, analyse and interpret the study data.

#### Intention-to-treat analysis (ITT)

The result of one study by Abraha et al. (2015) [21] indicates a statistically significant larger effect estimate for trials that report a deviation from the standard ITT approach with an overestimation of 20% (ROR 0.80; 95% CI: 0.69–0.94) than trials that applied the standard ITT approach. The evidence from this study combined the results of 202 clinical trials concerning pharmacological interventions. Subgroup analysis showed no difference between trials with subjective or objective or between placebo and non-placebo trials. After correction for in-between trial heterogeneity, a statistically significant result was retained [21].

ITT in RCTs comprises the analysis of patients in the treatment group to which they were randomly assigned, regardless of whether they have received the intervention of the comparison group or failed to complete the assigned treatment. ITT deviations may be treatment, post-baseline or baseline assessment or target condition related, or it may fall into multiple categories and may often not be clearly reported [21]. However, despite the apparent significant result, they are based on a limited number of pharmacological trials (N = 202) only. In addition, the trial design characteristic does not appear to lend itself as a suitable CQS criterion. Its relevance would be limited to RCTs with ITT analysis only; due to manifold and often unclear reported reasons for ITT deviation, no clear criterion in line with CQS principles could be formulated.

#### Allocation concealment

Meta-analysis results including two studies by Saltaji et al. (2018) [24] and Fenwick et al. (2008) [25] indicated a statistically significant larger effect estimate for trials with ‘inadequate’ or ‘unclear’ allocation concealment (dSMD 0.15; 95%CI: 0.03 to 0.28; I^2^ = 0%) compared to trials where allocation concealment was judged to be ‘adequate’. The evidence from both studies combined the results of 379 clinical, dental, oral and craniofacial trials.

Definitions for ‘adequate’, ‘inadequate’ and ‘unclear’ allocation concealment were based on Cochrane’s RoB tool (Version 1) [26]. Allocation concealment was considered ‘adequate’ if it was reported that the allocation sequence was kept in a locked computer file, translated into identical, coded, serially administered containers and/or sealed, opaque envelopes, together with the reassurance that the person who generated the sequence did not administer it. Allocation concealment was considered ‘inadequate’ if a transparent allocation procedure was used (e.g. alternation, use of patient data or an open random list). ‘Unclear’ allocation concealment was considered when no allocation concealment was reported in the text.

The current CQS–Criterion II requires that allocation concealment be in some form reported in the text [9]. It thus excludes all trials with ‘unclear’ allocation concealment, while corroborating trials with ‘adequate’ and ‘inadequate’ allocation concealment, without making any distinction between the two. In line with the identified evidence [24, 25], this criterion was reformulated as follows:

- ***Keeping the random allocation sequence in a locked computer file;***
- ***Translating the sequence into identical*, *coded*, *serially administered containers and/or sealed*, *opaque envelopes; and***
- ***Reassuring that the person who generated the sequence did not administer it*.**

Are in some form reported in the text (Yes = 1 / No = 0).

#### Multi-centre study design

Meta-analysis results including two studies by Bafeta et al. (2012) [27] and Dechartres et al. (2011) [28] indicated a statistically significant larger effect estimate for single-centre trials with an overestimation of 22% (ROR 0.78; 95%CI: 0.68 to 0.91; I^2^ = 56.6%) in comparison to multi-centre trials. The evidence from both studies combined the results of 292 pharmacological and non-pharmacological (specifically psychological or educational trials [27]).

While the study by Bafeta et al. (2012) included trials with continuous and mainly subjective outcomes [27], the study by Dechartres et al. (2011) included trials with binary outcomes and provided no further information concerning their subjective or objective nature [28]. Both studies showed an overall statistically significant larger effect estimate for single-centre trials, including pharmacological and non-pharmacological combined. However, subgroup analysis in the trial by Bafeta et al. (2012) for pharmacological and non-pharmacological trials, separately, found no statistically significant larger effect for either (dSMD -0.11; 95%CI: -0.29 to 0.018; p = 0.27 and dSMD –0.08; 95%CI: -0.18 to 0.01, respectively). In addition, both studies showed a smaller sample size for single-centre studies. Sensitivity analysis by Bafeta et al. (2012) revealed no statistically significant larger effect for single-centre studies after adjustment for sample size [27].

Due to the apparent discrepancy between overall and field specific results, possible differences between continuous and binary, as well as subjective and objective trial outcomes and a small-study effect as potential confounder of the overall results, no clear criterion in line with CQS principles can be formulated.

#### Sample size limit

Statistically significant effect estimate differences were established between trials with sample sizes of above and below 100, 500 and 1000 patients per intervention group.

Meta-analysis results including two studies by Zhang et al. (2013) [29] and Dechartres et al. (2013) [23] indicated a statistically significant larger effect estimate for trials with <100 patients per intervention group with an overestimation of 33% (ROR 0.68; 95%CI: 0.54 to 0.82; I^2^ = 80.2%) in comparison to trials with at least 100 patients. In addition, a further study by Nüesch et al. (2010) that could not be included in the meta-analysis also showed a statistically significant effect estimate difference (ES –0.21; 95%CI: -034 to –0.08) [22]. The evidence from both studies included in the meta-analysis combined the results of 919 trials that assess a wide range of therapeutic interventions and all subspecialties of critical care medicine.

A second meta-analysis, also including data from the studies by Dechartres et al. (2013) [23] and Papageorgiou et al. (2014) [30], did not establish a statistically significant effect estimate difference between trials with a sample size of below and above 200 patients (S2 File MA-ID10 Page 16), despite both studies indicating a statistically significant effect estimate difference each (ROR 0.76; 95%CI: 0.68 to 0.85 [23] and ROR 0.92; 95%CI: 0.87 to 0.98 [30]). The reason for the pooled non-significant difference may be due to high statistically heterogeneity between both studies (I^2^ = 88.6%), thus generating a very large-pooled CI in order to encompass all apparent variability. However, due to its non-significance, the pooled result was not considered for CQS extension.

In addition, the study by Dechartres et al. (2013) established statistically significant effect estimate differences between trials with a sample size of below and above 500 patients (ROR 0.81; 95% CI 0.74 to 0.89), as well as below and above 1000 patients (ROR 0.82; 95% CI 0.76 to 0.90) per intervention group. The evidence included the results of 434 and 301 trials respectively [23].

From the results of these studies, an increase in the effect size overestimation related to the decrease in sample size, with the highest for trials with fewer than 100 patients per intervention group was observed: 18% (<1000 patients); 19% (<500 patients) and 33% (<100 patients).

The current CQS–Criterion III requires that trials should have a sample size of at least 200 patients per intervention group [9]. In line with the identified evidence, this criterion was reformulated as follows:

### The sample size of any particular treatment group reported in the trial is not less than N = 100 (Yes = 1 / No = 0)

An overview of the extended CQS with its amended and added appraisal criteria is presented in Table 2.

**Table 2:** Appraisal criteria of the extended Composite Quality Score (CQS).

Criterion I	‘Randomisation’ for allocation to treatment groups is in some form reported in the text (Yes = 1 / No = 0)
Criterion II	(i) Keeping the random allocation sequence in a locked computer file; and (ii) Translating the sequence into identical, coded, serially administered containers and/or sealed, opaque envelopes; and (iii) Reassuring that the person who generated the sequence did not administer it are in some form reported in the text (Yes = 1 / No = 0)
Criterion III	Double-blinding or the blinding of at least two out of the three groups: trial participants; trial personnel and trial outcome assessors in some form reported in the text (Yes = 1 / No = 0)
Criterion IV	The sample size of any particular treatment group reported in the trial is not less than N = 100 (Yes = 1 / No = 0)

## Discussion

The aim of this study was to conduct a survey of current meta-epidemiological studies in order to identify trial design characteristics associated with statistically significant over- or underestimation of the true therapeutic effect, as basis for CQS extension. As result, one criterion concerning double-blinding was added as new criterion III. Criterion II (concerning allocation concealment) and criterion III (concerning sample size) of the original CQS version were amended and now form criterion II and IV respectively.

### Study limitations

Our study’s survey results depend greatly on the two systematic reviews by Page et al. (2016) and Dechartres et al. (2016) and thus shares their limitations [15, 16]. However, both reviews followed extensive, well-defined search strategies and included a large number of meta-epidemiological studies. For that reason, we believe their results to be comprehensive.

However, the main limitation of our study appears to be the reliance on meta-epidemiological research as basis for developing a trial appraisal tool. It has been argued that meta-epidemiological studies are only of observational nature and thus can only show correlation and not causation between the lack of trial design characteristics and trial over- or underestimation [31]. The observational nature is based on the fact that, in meta-epidemiological studies, exposures of interest cannot be independently manipulated, for example by randomisation, and that estimates of bias ascribed to one trial design characteristic may be confounded by differences in other trial characteristics that cannot be equally distributed between compared studies. For this reason, the lack of one trial design characteristic, such as lack of adequate random allocation, may lead to either an over- or underestimation of the true effect estimate, or to no deviation at all [32].

Additional limitations of meta-epidemiological studies may also include the fact that biases are unlikely to operate independently in trials, misclassification of trial design characteristics and publication (non-reporting) bias [32]. Against this background, Herbert (2020) suggested that meta-epidemiological studies might provide only little value in informing the design of clinical trials, particularly if they are unable to provide evidence that the lack of a trial design characteristic causes a particular bias [31].

Such a stance is indeed correct when adopted from a verification point of view. Verification is defined as hypothesis confirmation through supporting empirical facts [33]. From strong theoretical considerations, the hypothesis that a lack of a particular trial design characteristic causes trial over- or underestimation can be deduced [31]. However, when such hypothesis cannot be empirically tested because the only currently available format of meta-epidemiological research is unable to establish such causality, then supporting facts and consequently hypothesis verification are lacking and cannot be the basis for informing clinical trial design or appraisal.

However, from a falsification point of view, the current inability of meta-epidemiological research to establish such causality is of little importance. This view accepts that there is no possible situation in which a hypothesis can be verified by any particular set of observations but can always be falsified by a singular one [33, 34]. Consequently, when a hypothesis is compared with empirical facts from a set of currently available observations, such comparison constitutes a test. If the test outcome is negative, then empirical facts are shown to contradict the hypothesis, which in turn is then considered falsified. Such falsification provides sufficient reason not to accept the hypothesis for the time being. If the test outcome is positive, the hypothesis is considered corroborated. Corroboration does not mean justification that the hypothesis is true. It is only an indication that the hypothesis has for the time being passed the test and thus there is no current reason to reject it. Future tests may falsify the hypothesis, but as long as it remains corroborated, the hypothesis is capable of giving a good explanation of reality and does not conflict with empirical facts.

Against the background of the falsification point of view [33, 34], the results of our current survey of meta-epidemiological data are sufficient to be used as a basis for CQS extension.

### Study results

The extended CQS includes four criteria concerning generating and concealing of the random allocation sequence, double-blinding and sample size limit (Table 2). While the first three criteria appear in keeping with other trial appraisal methods such as Cochrane’s RoB tool [1, 26], the use of sample size as clinical trial appraisal criterion appears to be problematic.

It can be argued that any minimum sample size limit as trial appraisal criterion can never be precise and therefore will always be arbitrary [10]. Such a limit will always have to be research-field specific and needs to consider specific requirements for trial type, choice of trial outcome measure and minimum realistic difference between test and control intervention, as well as the choice for Type I and II error [9]. However, such considerations are limited to aspects concerning random error in trial design. Random error has been defined as the difference between the observed and the true value by chance, due to natural variations or imprecise measurement [35]. Such error affects trial precision, namely the reproducibility of results under the same conditions. To this regard, the probability of Type II–error (β) is of importance. The higher such probability, the higher the chance of a trial generating false negative results (the failure to show a treatment effect difference between test and control intervention when in reality there is one) [36]. Such probability may be reduced, based on power calculation, when a sufficiently large sample size is determined. A trial with a too low sample size may still yield statistically significant (p < 0.05) results, but only, if by chance, the observed difference in treatment effect is much larger than the real one. However, if the sample size is large enough in a trial (due to power calculation), then there is a high probability of establishing a true treatment effect difference as trial result, provided such difference exists in reality [37].

In contrast to random error, systematic error is the consistent difference between the observed and the true value. Such error affects not the precision but the accuracy of a clinical trial, namely how closely the observed trial results resemble the true ones. It has been observed that trials with smaller sample sizes show larger treatment effects than trials with larger sample sizes [38]. This ‘small-study effect’ appears not to be caused by random error (chance) but by systematic and consistent factors. These factors include publication and non-reporting bias [39], generally lower methodological trial quality [40, 41] and differences in the clinical heterogeneity between patients included in smaller versus larger trials [42]. The effect appears to be consistent across a wide range of therapeutic interventions and within a large number of trials, as the result of our investigation shows. Since the causes of the small-study effect are systematic, the effect is consistent and research-field independent. Therefore, the effect is not amendable by power calculation if such calculation establishes a samples size that is still considered as ‘small’. Based on the empirical findings of this investigation, a small sample size appears to be one that is below 100 patients per intervention group.

It may further be argued that a minimum sample size as trial appraisal criterion in systematic reviews may be erroneous, since results from trials that have passed all other CQS criteria could be pooled by meta-analysis in order to reach the required sample size threshold and, in such a case, a zero-score for the sample size criterion may be disregarded [10]. Such consideration is incorrect since the inclusion of small trials may divert the results of meta-analyses away from the true treatment effect size [23]. Against this background, it has been recommended that results from small trials as well as from meta-analyses including mainly small trials should be interpreted with caution [22, 23, 29] and that the results from larger trials and from meta-analyses of larger trials could be closer to the therapeutic truth [23].

Based on these considerations, there appears to be no reason for not including a sample size criterion (minimum sample size of 100 per intervention group) that follows a sufficient explanation of reality [38–41] and that is in agreement with current empirical facts [22, 23, 29] of the CQS.

### Application of the extended CQS (CQS-2)

Application of the CQS-2 includes binary trial rating per appraisal criterion with the scores 0 = invalid/falsified and score 1 = corroborated; multiplication of the single rating scores to an overall appraisal score and identification of trials with high bias risk, based on an overall 0-score [10]. The CQS-2 assures that a 0-score for any single criterion will nullify any gained 1-scores for other criteria and that the overall score will be zero. This is in keeping with the recognition that any single systematic or random error can invalidate clinical trial results [4]. In contrast, an overall 1-score does not reflect ‘low bias risk’ but only indicates that a trial has hitherto not failed any applied appraisal criterion, yet [10].

### Recommendations for further research

Currently, the CQS is still in development. It is recommended that further research should include an investigation of whether the CQS extension will have affected the very high inter-rater reliability of the first CQS version. In addition, trials from systematic reviews that have applied the 2^nd^ version of Cochrane’s RoB tool may be re-appraised using an extended CQS in order to compare whether systematic review conclusions and recommendations remain the same. Based on the results of these further investigations, an extended CQS may be piloted as part of the regular systematic review methodology for prospective, controlled clinical therapy trials.

## Conclusions

Our survey identified 38 meta-epidemiological studies. From these, seven trial design characteristics associated with statistically significant over- or underestimation of the true therapeutic effect were found. Based on these, one criterion concerning double-blinding was added and the original CQS criteria for concealing the random allocation sequence and for minimum sample size were amended.

## Supporting information

S1 File PRISMA checklist. (DOC)

S2 File (DOC)

S3 File PRISMA flow diagram. (DOC)
