# Supplementary material for: Extension of the Composite Quality Score (CQS) as an appraisal tool for prospective, controlled clinical therapy trials–A systematic review of meta-epidemiological evidence
Source: PLoS One. 2022 Dec 30;17(12):e0279645. doi: 10.1371/journal.pone.0279645 (PMC9803107; doi:10.1371/journal.pone.0279645)
Supplement: S2 File — (DOC) [file pone.0279645.s002.doc]

**Identification of studies via databases and registers**

**Identification of studies via other methods**

Records identified from*:

Databases

-PubMed (n = 309)

-Embase (n = 251)

-Page et al (n = 1)

Reference checks

-Page et al., 2016 (n = 17)

-Dechartres et al., 2016 (n = 15)

Records removed *before screening*:

Duplicate/not relevant records removed (n = 534 )

Records identified from:

Websites (n = 0 )

Organisations (n = 0 )

Citation searching (n = 0 )

etc.

**Identification**

Records excluded** (n = 21 )

Reason 1 (n = 14)

Reason 2 (n = 1)

Reason 3 (n = 4)

Reason 4 (n = 1)

Reason 5 (n = 1)

Records screened

(n = 59)

Reports sought for retrieval

(n = 38)

Reports not retrieved

(n = 0)

Reports sought for retrieval

(n = 0)

Reports not retrieved

(n = 0)

**Screening**

Reports assessed for eligibility

(n = 38 )

Reports excluded:

Reason 1 (n = 0)

Reason 2 (n = 0)

Reason 3 (n = 0)

Reports assessed for eligibility

(n = 0)

Reports excluded:

Reason 1 (n = 0)

Reason 2 (n = 0)

Reason 3 (n = 0)

etc.

Studies included in review

(n = 38)

Reports of included studies

(n = 38)

**Included**

*Consider, if feasible to do so, reporting the number of records identified from each database or register searched (rather than the total number across all databases/registers).

**If automation tools were used, indicate how many records were excluded by a human and how many were excluded by automation tools.

*From:*  Page MJ, McKenzie JE, Bossuyt PM, Boutron I, Hoffmann TC, Mulrow CD, et al. The PRISMA 2020 statement: an updated guideline for reporting systematic reviews. BMJ 2021;372:n71. doi: 10.1136/bmj.n71. For more information, visit: <http://www.prisma-statement.org/>
