# Supplementary material for: Extension of the Composite Quality Score (CQS) as an appraisal tool for prospective, controlled clinical therapy trials–A systematic review of meta-epidemiological evidence
Source: PLoS One. 2022 Dec 30;17(12):e0279645. doi: 10.1371/journal.pone.0279645 (PMC9803107; doi:10.1371/journal.pone.0279645)
Supplement: S3 File — (DOC) [file pone.0279645.s003.doc]

Extension of the Composite Quality Score (CQS) as an appraisal tool for prospective, controlled clinical therapy trials – A systematic review of meta-epidemiological evidence (Additional file)

Steffen Mickenautsch^1,2,3*^, Stefan Rupf^4^, Ivana Miletić^5^, Veerasamy Yengopal^1^

^1^Faculty of Dentistry, University of the Western Cape, Francie van Zijl Avenue, Tygerberg/Cape Town, 7505, South Africa.

^2^ Honorary/Department of Community Dentistry, School of Oral Health Sciences, Faculty of Health Sciences, University of the Witwatersrand, 7 York Rd., Parktown/Johannesburg 2193, South Africa.

^3^Review Center For Health Science Research, 84 Concorde Road East, Bedfordview/Johannesburg, 2008, South Africa.

^4^Chair of Synoptic Dentistry, Saarland University, Building 73, 66421, Homburg, Germany.

^5^Department of Endodontics and Restorative Dentistry, School of Dental Medicine, University of Zagreb, Gunduliceva 5, 10 000, Zagreb, Croatia.

- List of excluded studies Page 2
- List of included studies Page 3
- Extracted datasets Page 6
- Excluded datasets Page 11
- Datasets for meta-analysis Page 14
- Meta-analysis results Page 15
- Included single datasets Page 18

# Excluded studies

| Study ID | From source | Reference | Reason for exclusion |
| --- | --- | --- | --- |
| 25 | Reference check:  Page et al. [84] / Studies excluded by authors | Als-Nielsen B, Chen W, Gluud C, Kjaergard LL. Association of funding and conclusions in randomized drug trials: a reflection of treatment effect or adverse events? JAMA. 2003 Aug 20;290(7):921-8. | No computed comparison result related to a trial design characteristic extractable |
| 27 |  | Bes-Rastrollo M, Schulze MB, Ruiz-Canela M, Martinez-Gonzalez MA. Financial conflicts of interest and reporting bias regarding the association between sugar-sweetened beverages and weight gain: a systematic review of systematic reviews. PLoS Med. 2013 Dec;10(12):e1001578; dicsussion e1001578. | No computed comparison result related to a trial design characteristic extractable |
| 30 |  | MacLehose RR, Reeves BC, Harvey IM, Sheldon TA, Russell IT, Black AM. A systematic review of comparisons of effect sizes derived from randomised and non-randomised studies. Health Technol Assess. 2000;4(34):1-154. | Comparison related to differences outside prospective, clinical controlled study design characteristics |
| 32 |  | Munder T, Brütsch O, Leonhart R, Gerger H, Barth J. Researcher allegiance in psychotherapy outcome research: an overview of reviews. Clin Psychol Rev. 2013 Jun;33(4):501-11. | No computed comparison result related to a trial design characteristic extractable |
| 34 |  | Panagiotou OA, Contopoulos-Ioannidis DG, Ioannidis JP. Comparative effect sizes in randomised trials from less developed and more developed countries: meta-epidemiological assessment. BMJ. 2013 Feb 12;346:f707. | No computed comparison result related to a trial design characteristic extractable |
| 36 |  | Papageorgiou SN, Tsiranidou E, Antonoglou GN, Deschner J, Jäger A. Choice of effect measure for meta-analyses of dichotomous outcomes influenced the identified heterogeneity and direction of small-study effects. J Clin Epidemiol. 2015 May;68(5):534-41. | Not relevant. |
| 37 |  | Peura PK, Martikainen JA, Purmonen TT, Turunen JH. Sponsorship-related outcome selection bias in published economic studies of triptans: systematic review. Med Decis Making. 2012 Mar-Apr;32(2):237-45. | No computed comparison result related to a trial design characteristic extractable |
| 38 |  | Saquib N, Saquib J, Ioannidis JP. Practices and impact of primary outcome adjustment in randomized controlled trials: meta-epidemiologic study. BMJ. 2013 Jul 12;347:f4313. | No computed comparison result related to a trial design characteristic extractable |
| 39 |  | Seegers V, Trinquart L, Boutron I, Ravaud P. Comparison of treatment effect estimates for pharmacological randomized controlled trials enrolling older adults only and those including adults: a meta-epidemiological study. PLoS One. 2013 May 28;8(5):e63677. | Not relevant. |
| 40 |  | Sichieri R, Cunha DB. Unbalanced baseline in school-based interventions to prevent obesity: adjustment can lead to bias - a systematic review. Obes Facts. 2014;7(4):221-32. | No computed comparison result related to a trial design characteristic extractable |
| 53 | PubMed Search | Hulshof TA, Zuidema SU, Gispen-de Wied CC, Luijendijk HJ. Run-in periods and clinical outcomes of antipsychotics in dementia: A meta-epidemiological study of placebo-controlled trials. Pharmacoepidemiol Drug Saf. 2020 Feb;29(2):125-133. | No computed comparison result related to a trial design characteristic extractable |
| 55 |  | de Almeida MO, Saragiotto BT, Maher C, Costa LOP. Allocation Concealment and Intention-To-Treat Analysis Do Not Influence the Treatment Effects of Physical Therapy Interventions in Low Back Pain Trials: a Meta-epidemiologic Study. Arch Phys Med Rehabil. 2019 Jul;100(7):1359-1366. | No computed comparison result related to a trial design characteristic extractable |
| 58 |  | Dechartres A, Atal I, Riveros C, Meerpohl J, Ravaud P. Association Between Publication Characteristics and Treatment Effect Estimates: A Meta-epidemiologic Study. Ann Intern Med. 2018 Sep 18;169(6):385-393. | Not relevant. |
| 65 |  | Christensen AW, Tarp S, Furst DE, Døssing A, Amris K, Bliddal H, Taylor PC, Christensen R. Most Trial Eligibility Criteria and Patient Baseline Characteristics Do Not Modify Treatment Effect in Trials Using Targeted Therapies for Rheumatoid Arthritis: A Meta-Epidemiological Study. PLoS One. 2015 Sep 11;10(9):e0136982. | Not relevant. |
| 67 |  | Koletsi D, Spineli LM, Lempesi E, Pandis N. Risk of bias and magnitude of effect in orthodontic randomized controlled trials: a meta-epidemiological review. Eur J Orthod. 2016 Jun;38(3):308-12. | No computed comparison result related to a trial design characteristic extractable |
| 69 | Reference check:  Dechartres A, Trinquart L, Faber T, Ravaud P. Empirical evaluation of which trial characteristics are associated with treatment effect estimates. J Clin Epidemiol. 2016 Sep;77:24-37. | Tierney JF, Stewart LA. Investigating patient exclusion bias in meta-analysis. Int J Epidemiol. 2005 Feb;34(1):79-87. | No computed comparison result related to a trial design characteristic extractable |
| 70 |  | Bassler D, Briel M, Montori VM, Lane M, Glasziou P, Zhou Q, Heels-Ansdell D, Walter SD, Guyatt GH; STOPIT-2 Study Group, Flynn DN, Elamin MB, Murad MH, Abu Elnour NO, Lampropulos JF, Sood A, Mullan RJ, Erwin PJ, Bankhead CR, Perera R, Ruiz Culebro C, You JJ, Mulla SM, Kaur J, Nerenberg KA, Schünemann H, Cook DJ, Lutz K, Ribic CM, Vale N, Malaga G, Akl EA, Ferreira-Gonzalez I, Alonso-Coello P, Urrutia G, Kunz R, Bucher HC, Nordmann AJ, Raatz H, da Silva SA, Tuche F, Strahm B, Djulbegovic B, Adhikari NK, Mills EJ, Gwadry-Sridhar F, Kirpalani H, Soares HP, Karanicolas PJ, Burns KE, Vandvik PO, Coto-Yglesias F, Chrispim PP, Ramsay T. Stopping randomized trials early for benefit and estimation of treatment effects: systematic review and meta-regression analysis. JAMA. 2010 Mar 24;303(12):1180-7. | No computed comparison result related to a trial design characteristic extractable |
| 77 |  | Hempel S, Suttorp MJ, Miles JNV, Wang Z, Maglione M, Morton S, Johnsen B, Valentine D, Shekelle PG. Empirical Evidence of Associations Between Trial Quality and Effect Size [Internet]. Rockville (MD): Agency for Healthcare Research and Quality (US); 2011 Jun. Report No.: 11-EHC045-EF. | Older study version. |
| 78 |  | Jacobs WC, Kruyt MC, Verbout AJ, Oner FC. Effect of methodological quality measures in spinal surgery research: a metaepidemiological study. Spine J. 2012 Apr;12(4):339-48. | No computed comparison result related to a trial design characteristic extractable |
| 83 |  | Turner RM, Bird SM, Higgins JP. The impact of study size on meta-analyses: examination of underpowered studies in Cochrane reviews. PLoS One. 2013;8(3):e59202. | No computed comparison result related to a trial design characteristic extractable |
| 66 |  | Armijo-Olivo S, Saltaji H, da Costa BR, Fuentes J, Ha C, Cummings GG. What is the influence of randomisation sequence generation and allocation concealment on treatment effects of physical therapy trials? A meta-epidemiological study. BMJ Open. 2015 Sep 3;5(9):e008562. | Duplication (same as Study ID 12) |

**Included studies**

| From source | Study ID | Citation | Dataset ID |
| --- | --- | --- | --- |
|  |  |  |  |
| Direct data extraction | 84 | Page MJ, Higgins JP, Clayton G, Sterne JA, Hróbjartsson A, Savović J. Empirical Evidence of Study Design Biases in Randomized Trials: Systematic Review of Meta-Epidemiological Studies. PLoS One. 2016 Jul 11;11(7):e0159267. | 01 |
|  |  |  | 02 |
|  |  |  | 03 |
|  |  |  | 04 |
|  |  |  | 05 |
|  |  |  | 06 |
|  |  |  | 07 |
|  |  |  | 09 |
|  |  |  | 10 |
|  |  |  | 11 |
|  |  |  | 12 |
| Reference check:  Page et al. [84] / Studies included by authors with non RoB tool related comparisons | 11 | Abraha I, Cherubini A, Cozzolino F, De Florio R, Luchetta ML, Rimland JM, Folletti I, Marchesi M, Germani A, Orso M, Eusebi P, Montedori A. Deviation from intention to treat analysis in randomised trials and treatment effect estimates: meta-epidemiological study. BMJ. 2015 May 27;350:h2445. | 08 |
|  |  |  | 13 |
|  | 12 | Armijo-Olivo S, Saltaji H, da Costa BR, Fuentes J, Ha C, Cummings GG. What is the influence of randomisation sequence generation and allocation concealment on treatment effects of physical therapy trials? A meta-epidemiological study. BMJ Open. 2015 Sep 3;5(9):e008562. | 14 |
|  |  |  | 15 |
| Reference check:  Page et al. [84] / Studies excluded by authors | 26 | Bafeta A, Dechartres A, Trinquart L, Yavchitz A, Boutron I, Ravaud P. Impact of single centre status on estimates of intervention effects in trials with continuous outcomes: meta-epidemiological study. BMJ. 2012 Feb 14;344:e813. | 16 |
|  | 29 | Lonjon G, Boutron I, Trinquart L, Ahmad N, Aim F, Nizard R, Ravaud P. Comparison of treatment effect estimates from prospective nonrandomized studies with propensity score analysis and randomized controlled trials of surgical procedures. Ann Surg. 2014 Jan;259(1):18-25. | 17 |
|  | 35 | Papageorgiou SN, Antonoglou GN, Tsiranidou E, Jepsen S, Jäger A. Bias and small-study effects influence treatment effect estimates: a meta-epidemiological study in oral medicine. J Clin Epidemiol. 2014 Sep;67(9):984-92. | 18 |
|  |  |  | 19 |
|  |  |  | 20 |
|  | 41 | Smaïl-Faugeron V, Fron-Chabouis H, Courson F, Durieux P. Comparison of intervention effects in split-mouth and parallel-arm randomized controlled trials: a meta-epidemiological study. BMC Med Res Methodol. 2014 May 11;14:64. | 21 |
|  |  |  | 22 |
|  | 42 | Zhang Z, Xu X, Ni H. Small studies may overestimate the effect sizes in critical care meta-analyses: a meta-epidemiological study. Crit Care. 2013 Jan 9;17(1):R2. | 23 |
| PubMed Search | 43 | Saltaji H, Armijo-Olivo S, Cummings GG, Amin M, Major PW, da Costa BR, Flores-Mir C. Influence of Sponsorship Bias on Treatment Effect Size Estimates in Randomized Trials of Oral Health Interventions: A Meta-epidemiological Study. J Evid Based Dent Pract. 2021 Jun;21(2):101544. | 24 |
|  | 44 | Armijo-Olivo S, da Costa BR, Ha C, Saltaji H, Cummings GG, Fuentes J. Are biases related to attrition, missing data, and the use of intention to treat related to the magnitude of treatment effects in physical therapy trials? A meta-epidemiological study. Am J Phys Med Rehabil. 2021 Jul 3. doi: 10.1097/PHM.0000000000001837. | 25 |
|  |  |  | 26 |
|  |  |  | 27 |
|  | 45 | Wang Z, Alahdab F, Farah M, Seisa M, Firwana M, Rajjoub R, Saadi S, Jawaid T, Nayfeh T, Murad MH. Association of study design features and treatment effects in trials of chronic medical conditions: a meta-epidemiological study. BMJ Evid Based Med. 2021 Jul 1:bmjebm-2021-111667. | 28 |
|  |  |  | 29 |
|  |  |  | 30 |
|  |  |  | 31 |
|  |  |  | 32 |
|  |  |  | 33 |
|  |  |  | 34 |
|  | 46 | Mostazir M, Taylor G, Henley WE, Watkins ER, Taylor RS. Per protocol analyses produced larger treatment effect sizes than intention to treat: a meta-epidemiological study. J Clin Epidemiol. 2021 Jun 20;138:12-21. | 35 |
|  | 47 | Martin GL, Trioux T, Gaudry S, Tubach F, Hajage D, Dechartres A. Association Between Lack of Blinding and Mortality Results in Critical Care Randomized Controlled Trials: A Meta-Epidemiological Study. Crit Care Med. 2021 Apr 30. doi: 10.1097/CCM.0000000000005065. | 36 |
|  | 49 | Fuentes J, Armijo-Olivo S, da Costa BR, Ha C, Saltaji H, Arenti C, Negrini S, Cummings GG. Does Type of Sponsorship of Randomized Controlled Trials Influence Treatment Effect Size Estimates in Rehabilitation: A Meta-Epidemiological Study. Am J Phys Med Rehabil. 2020 Oct;99(10):909-916. | 37 |
|  | 50 | Haring R, Ghannad M, Bertizzolo L, Page MJ. No evidence found for an association between trial characteristics and treatment effects in randomized trials of testosterone therapy in men: a meta-epidemiological study. J Clin Epidemiol. 2020 Jun;122:12-19. | 38 |
|  |  |  | 39 |
|  |  |  | 40 |
|  |  |  | 41 |
|  |  |  | 42 |
|  |  |  | 43 |
|  |  |  | 44 |
|  |  |  | 45 |
|  | 51 | Moustgaard H, Clayton GL, Jones HE, Boutron I, Jørgensen L, Laursen DRT, Olsen MF, Paludan-Müller A, Ravaud P, Savovic J, Sterne JAC, Higgins JPT, Hróbjartsson A. Impact of blinding on estimated treatment effects in randomised clinical trials: meta-epidemiological study. BMJ. 2020 Jan 21;368:l6802. doi: 10.1136/bmj.l6802. Erratum in: BMJ. 2020 Feb 5;368:m358. | 46 |
|  |  |  | 47 |
|  |  |  | 48 |
|  |  |  | 49 |
|  | 52 | Armijo-Olivo S, Dennett L, Arienti C, Dahchi M, Arokoski J, Heinemann AW, Malmivaara A. Blinding in Rehabilitation Research: Empirical Evidence on the Association Between Blinding and Treatment Effect Estimates. Am J Phys Med Rehabil. 2020 Mar;99(3):198-209. | 50 |
|  |  |  | 51 |
|  | 54 | Ewald H, Ioannidis JPA, Ladanie A, Mc Cord K, Bucher HC, Hemkens LG. Nonrandomized studies using causal-modeling may give different answers than RCTs: a meta-epidemiological study. J Clin Epidemiol. 2020 Feb;118:29-41. | 52 |
|  | 56 | Mathes T, Klaßen P, Pieper D. No differences were found between effect estimates from conventional and registry-based randomized controlled trials. J Clin Epidemiol. 2019 Jan;105:80-91. | 53 |
|  | 57 | Janiaud P, Cristea IA, Ioannidis JPA. Industry-funded versus non-profit-funded critical care research: a meta-epidemiological overview. Intensive Care Med. 2018 Oct;44(10):1613-1627. | 54 |
|  | 59 | Papageorgiou SN, Xavier GM, Cobourne MT, Eliades T. Registered trials report less beneficial treatment effects than unregistered ones: a meta-epidemiological study in orthodontics. J Clin Epidemiol. 2018 Aug;100:44-52. | 55 |
|  | 60 | Savovic J, Turner RM, Mawdsley D, Jones HE, Beynon R, Higgins JPT, Sterne JAC. Association Between Risk-of-Bias Assessments and Results of Randomized Trials in Cochrane Reviews: The ROBES Meta-Epidemiologic Study. Am J Epidemiol. 2018 May 1;187(5):1113-1122. | 56 |
|  |  |  | 57 |
|  |  |  | 58 |
|  |  |  | 59 |
|  | 61 | Saltaji H, Armijo-Olivo S, Cummings GG, Amin M, da Costa BR, Flores-Mir C. Impact of Selection Bias on Treatment Effect Size Estimates in Randomized Trials of Oral Health Interventions: A Meta-epidemiological Study. J Dent Res. 2018 Jan;97(1):5-13. | 60 |
|  |  |  | 61 |
|  |  |  | 62 |
|  | 62 | Dechartres A, Ravaud P, Atal I, Riveros C, Boutron I. Association between trial registration and treatment effect estimates: a meta-epidemiological study. BMC Med. 2016 Jul 4;14(1):100. | 63 |
|  |  |  | 64 |
|  |  |  | 65 |
|  | 63 | Armijo-Olivo S, Fuentes J, da Costa BR, Saltaji H, Ha C, Cummings GG. Blinding in Physical Therapy Trials and Its Association with Treatment Effects: A Meta-epidemiological Study. Am J Phys Med Rehabil. 2017 Jan;96(1):34-44. | 66 |
|  |  |  | 67 |
|  |  |  | 68 |
|  |  |  | 69 |
|  |  |  | 70 |
|  | 64 | Papageorgiou SN, Koretsi V, Jäger A. Bias from historical control groups used in orthodontic research: a meta-epidemiological study. Eur J Orthod. 2017 Feb;39(1):98-105. | 71 |
|  | 68 | Papageorgiou SN, Xavier GM, Cobourne MT. Basic study design influences the results of orthodontic clinical investigations. J Clin Epidemiol. 2015 Dec;68(12):1512-22. | 72 |
|  |  |  | 73 |
|  |  |  | 74 |
|  | 85 | To MS, Di Ubaldo LJ, Wells AJ, Jukes A. Absence of small study effects in neurosurgical meta-analyses: A meta-epidemiological study. J Clin Neurosci. 2021 Nov;93:137-140. | 106 |
| Reference check:  Dechartres A, Trinquart L, Faber T, Ravaud P. Empirical evaluation of which trial characteristics are associated with treatment effect estimates. J Clin Epidemiol. 2016 Sep;77:24-37. | 71 | Dechartres A, Boutron I, Trinquart L, Charles P, Ravaud P. Single-center trials show larger treatment effects than multicenter trials: evidence from a meta-epidemiologic study. Ann Intern Med. 2011 Jul 5;155(1):39-51. | 75 |
|  | 72 | Dechartres A, Trinquart L, Boutron I, Ravaud P. Influence of trial sample size on treatment effect estimates: meta-epidemiological study. BMJ. 2013 Apr 24;346:f2304. | 76 |
|  |  |  | 77 |
|  |  |  | 78 |
|  |  |  | 79 |
|  |  |  | 80 |
|  | 73 | Nüesch E, Trelle S, Reichenbach S, Rutjes AW, Tschannen B, Altman DG, Egger M, Jüni P. Small study effects in meta-analyses of osteoarthritis trials: meta-epidemiological study. BMJ. 2010 Jul 16;341:c3515. | 81 |
|  | 75 | Fenwick J, Needleman IG, Moles DR. The effect of bias on the magnitude of clinical outcomes in periodontology: a pilot study. J Clin Periodontol. 2008 Sep;35(9):775-82. | 82 |
|  |  |  | 83 |
|  |  |  | 84 |
|  |  |  | 85 |
|  | 76 | Hempel S, Miles J, Suttorp MJ, Wang Z, Johnsen B, Morton S, Perry T, Valentine D, Shekelle PG. Detection of Associations Between Trial Quality and Effect Sizes [Internet]. Rockville (MD): Agency for Healthcare Research and Quality (US); 2012 Jan. Report No.: 12-EHC010-EF.  . | 86 |
|  |  |  | 87 |
|  |  |  | 88 |
|  |  |  | 89 |
|  |  |  | 90 |
|  |  |  | 91 |
|  |  |  | 92 |
|  | 79 | Khan KS, Daya S, Collins JA, Walter SD. Empirical evidence of bias in infertility research: overestimation of treatment effect in crossover trials using pregnancy as the outcome measure. Fertil Steril. 1996 May;65(5):939-45. | 93 |
|  |  |  | 94 |
|  |  |  | 95 |
|  |  |  | 96 |
|  | 80 | Lathyris DN, Trikalinos TA, Ioannidis JP. Evidence from crossover trials: empirical evaluation and comparison against parallel arm trials. Int J Epidemiol. 2007 Apr;36(2):422-30. | 97 |
|  | 81 | Mhaskar R, Djulbegovic B, Magazin A, Soares HP, Kumar A. Published methodological quality of randomized controlled trials does not reflect the actual quality assessed in protocols. J Clin Epidemiol. 2012 Jun;65(6):602-9. | 98 |
|  |  |  | 99 |
|  | 82 | Moher D, Cook DJ, Jadad AR, Tugwell P, Moher M, Jones A, Pham B, Klassen TP. Assessing the quality of reports of randomised trials: implications for the conduct of meta-analyses. Health Technol Assess. 1999;3(12):i-iv, 1-98. | 100 |
|  | 74 | Savović J, Jones HE, Altman DG, Harris RJ, Jüni P, Pildal J, Als-Nielsen B, Balk EM, Gluud C, Gluud LL, Ioannidis JP, Schulz KF, Beynon R, Welton NJ, Wood L, Moher D, Deeks JJ, Sterne JA. Influence of reported study design characteristics on intervention effect estimates from randomized, controlled trials. Ann Intern Med. 2012 Sep 18;157(6):429-38. | 103 |
|  |  |  | 104 |
|  |  |  | 105 |

# Extracted datasets

Total number = 104

Number of extracted datasets = 104

Number of excluded datasets = 77

Number of accepted datasets = 27

Number of datasets for meta-analyses= 21

Number of possible

meta-analyses = 10

Number of accepted single

datasets = 6

Excluded = 2

Included = 8

**List of extracted datasets**

# Abbreviations

| A = Adequate;  AC = Allocation concealment;  AT = Incomplete outcome data, withdrawals, losses to follow-up;  BLI = Blinding (no further specifications)  BO = Observer blinding;  BP = Patients blinding;  BR = Block randomisation;  BS = Personnel blinding;  BST = Statistician blinding  BSL = Baseline imbalance;  CI = Confidence interval  CO = Confounder adjustment  COI = Trialists with conflict of interest  CRO = Crossover design;  CrI = Credibility interval | DB = Double blinding;  DR = deviation from the standard approach reported;  DS = Dataset  dSMD = Differences in Standardized Mean Differences;  ES = Effect size;  EV = Estimate value;  FO = Follow-up;  FPF = For-profit funding source;  H = High bias risk;  HIST = Use of historic (untreated) control group;  IDC = Incomplete outcome data control;  IN = Inadequate;  IND = Industry funded;  ITT = Intention-to-treat analysis; | L = Low bias risk;  LCL = Lower confidence limit  LTF = Drop-out rate >20;  MA = Meta-analysis;  MC = Multi-centre study;  NO = standard approach not reported;  NR = Not reported;  PA = Parallel group study design;  PAD = Trials registered after primary completion date;  PCD = Trials registered before primary completion date;  PP = Per-protocol analysis;  PR = Prospectively registered trials  PRO = Prospective trials;  REG = Trial protocol registered  RET = Retrospective trials; | ROR = Ratio of Odds Ratios;  RL = One randomised arm at least x2 as large as the other;  RR = Retrospectively registered trials;  RS = Random sequence generation;  SPO = Sponsorship bias risk;  SR = Selective outcome reporting;  SS = Sample size  SS1 = Sample size >100 patients;  SS2 = Sample size >200 patients;  SS3 = Sample size >50 patients;  SS4 = Sample size >500 patients;  SS5 = Sample size >1000 patients;  TO = Type of outcomes (binary = 1 / continuous = 2);  TS = Trial stopped early;  UN = Unclear; | UCL = Upper confidence limit  UR = Un(not) registered trials;  YES = Standard approach reported; |
| --- | --- | --- | --- | --- |

| Dataset ID | Trial design characteristic investigated | Comparison type | Number of included | | | Type of estimate | Type of estimate precision measure | Estimate value (EV) | Type of heterogeneity estimate | Type of heterogeneity precision measure | Heterogeneity value | Direction of effect for over-estimation |
| --- | --- | --- | --- | --- | --- | --- | --- | --- | --- | --- | --- | --- |
|  |  |  | ME | MA | Trials |  |  |  |  |  |  |  |
| 01 | Random allocation | RS (IN/UN vs A) | 24 | NR | NR | ROR | 95%CI | 0.93 (0.86 to 0.99) | I^2^ | NR | 0% | EV < 1 |
| 02 | Allocation concealment | AC (IN/UN vs A) | 24 | NR | NR | ROR | 95%CI | 0.90 (0.84 to 0.97) | I^2^ | NR | 28% | EV < 1 |
| 03 | Baseline imbalance | BSL (YES vs No) | 24 | NR | NR | ROR | 95%CI | 1.03 (0.89 to 1.19) | I^2^ | NR | 0% | EV < 1 |
| 04 | Confounder adjustment | CO (NO vs YES) | 24 | NR | NR | ROR | 95%CI | 0.96 (0.79 to 1.23) | NR | NR | NR | EV < 1 |
| 05 | Block randomisation | BR (NO vs YES) | 24 | NR | NR | dSMD | 95%CI | -0.18 (-0.47 to 0.11) | NR | NR | NR | EV < 0 |
| 06 | Patients blinding | BP (NO/UN vs Yes) | 24 | NR | NR | ROR | 95%CI | 0.92 (0.81 to 1.04) | I^2^ | NR | 0% | EV < 1 |
| 07 | Personnel blinding | BS (NO/UN vs Yes) | 24 | NR | NR | ROR | 95%CI | 1.00 (0.86 to 1.16) | I^2^ | NR | 0% | EV < 1 |
| 08 | Intention-to-treat analysis | ITT (DR vs YES) | 24 | NR | 202 | ROR | 95%CI | 0.80 (0.69 to 0.94) | NR | NR | NR | EV < 1 |
| 09 | Drop-out rate | LTF (YES vs No) | 24 | NR | NR | ROR | 95%CI | 1.07 (0.92 to 1.25) | NR | NR | NR | EV < 1 |
| 10 | Observer blinding | BO (NO/UN vs Yes) | 24 | NR | NR | ROR | 95%CI | 1.01 (0.90 to 1.13) | I^2^ | NR | 0% | EV < 1 |
| 11 | Double blinding | DB (NO/UN vs Yes) | 24 | NR | NR | ROR | 95%CI | 0.77 (0.61 to 0.93) | NR | NR | NR | EV < 1 |
| 12 | Selective outcome reporting | SR (YES/UN vs A} | 24 | NR | NR | ROR | 95%CI | 0.71 (0.43 to 1.19) | NR | NR | NR | EV < 1 |
| 13 | Intention-to-treat analysis | ITT (NO vs DR) | NR | 50 | 226 | ROR | 95%CI | 0.92 (0.70 to 1.23) | τ^2^ | NR | 0.57 | EV < 1 |
| 14 | Random allocation | RS (IN vs A) | NR | 43 | 393 | ES | 95%CI | 0.02 (-0.12 to 0.15) | NR | NR | NR | EV < 0 |
| 15 | Allocation concealment | AC (IN vs A) | NR | 43 | 393 | ES | 95%CI | 0.12 (-0.06 to 0.30) | NR | NR | NR | EV < 0 |
| 16 | Multicenter study | MC (NO vs YES) | NR | 26 | 292 | dSMD | 95%CI | -0.09 (-0.17 to –0.01) | I^2^ | NR | 0% | EV < 0 |
| 17 | Random allocation | RS (NO vs YES) | NR |  | 164 | ROR | 95%CI | 0.83 (0.65 to 1.04) | I^2^ | NR | 0% | EV < 1 |
| 18 | Sample size | SS2 (NO vs YES) | NR | 62 | 619 | ROR | 95%CI | 0.92 (0.87 to 0.98) | I^2^ | 95%CI | 0% (0 to 25%) | EV < 1 |
| 19 | Random allocation | RS (NO vs YES) | NR | 24 | 246 | ROR | 95%CI | 0.94 (0.77 to 1.16) | I^2^ | 95%CI | 0% (0 to 40%) | EV < 1 |
| 20 | One randomised arm | RL (YES vs NO) | NR | 36 | 443 | ROR | 95%CI | 0.99 (0.91 to 1.08) | I^2^ | 95%CI | 0% (0 to 34%) | EV < 1 |
| 21 | Parallel group study design | PA (NO vs YES) | NR | 15 | 56 | ROR | 95%CI | 0.96 (0.52 to 1.80) | I^2^ | 95%CI | 50% (9 to 80%) | EV < 1 |
| 22 | Parallel group study design | PA (NO vs YES) | NR | 19 | 56 | dSMD | 95%CI | 0.08 (-0.14 to 0.30) | I^2^ | 95%CI | 56% (21 to 82%) | EV < 0 |
| 23 | Sample size | SS1 (NO vs YES) | NR | 27 | 317 | ROR | 95%CI | 0.60 (0.53 to 0.68) | I^2^ | NR | 50.3% | EV < 1 |
| 24 | Sponsorship bias risk | SPO (H/UN vs L) | NR | 64 | 540 | ES | 95%CI | 0.10 (0.02 to 0.19) | NR | NR | NR | EV > 0 |
| 25 | Incomplete outcome data control | IDC (IN vs A) | NR | 40 | 380 | ES | 95%CI | -0.11 (-0.20 to -0.01) | NR | NR | NR | EV > 0 |
| 26 | Intention-to-treat analysis | ITT (N/UN vs YES) | NR | 37 | 359 | ES | 95%CI | -0.04 (-0.14 to 0.06) | NR | NR | NR | EV > 0 |
| 27 | Drop-out rate | LTF (YES vs No) | NR | NR | NR | ES | 95%CI | -0.13 (-0.22 to –0.04) | NR | NR | NR | EV > 0 |
| 28 | Random allocation | RS (IN/UN vs A) | NR | NR | NR | ROR | 95%CI | 1.04 (0.93 to 1.16) | NR | NR | NR | EV < 1 |
| 29 | Allocation concealment | AC (IN/UN vs A) | NR | NR | NR | ROR | 95%CI | 1.10 (0.93 to 1.16) | NR | NR | NR | EV < 1 |
| 30 | Observer blinding | BO (NO/UN vs Yes) | NR | NR | NR | ROR | 95%CI | 0.97 (0.86 to 1.08) | NR | NR | NR | EV < 1 |
| 31 | Incomplete outcome data control | IDC (IN/UN vs A) | NR | NR | NR | ROR | 95%CI | 1.11 (0.99 to 1.24) | NR | NR | NR | EV < 1 |
| 32 | Multicenter study | MC (NO vs YES) | NR | NR | NR | ROR | 95%CI | 1.04 (0.93 to 1.16) | NR | NR | NR | EV < 1 |
| 33 | Trial stopped early | TS (YES/UN vs NO) | NR | NR | NR | ROR | 95%CI | 0.93 (0.78 to 1.12) | NR | NR | NR | EV < 1 |
| 34 | For-profit funding source | FPF (YES vs NO) | NR | NR | NR | ROR | 95%CI | 0.97 (0.85 to 1.11) | NR | NR | NR | EV < 1 |
| 35 | Intention-to-treat analysis | ITT (PP vs YES) | NR | NR | 156 | ROR | 95%CI | 1.02 (1.00 to 1.04) | NR | NR | NR | EV > 1 |
| 36 | Double blinding | DB (NO/UN vs Yes) | NR | 36 | 467 | ROR | 95%CI | 0.91 (0.84 to 0.99) | I^2^ | NR | 0% | EV < 1 |
| 37 | Sponsorship bias risk | SPO (H/UN vs L) | NR | 43 | 393 | ES | 95%CI | 0.15 (-0.03 to 0.33) | I^2^ | NR | 63.4% | EV > 0 |
| 38 | Sample size | SS3 (NO vs YES) | NR | NR | NR | ROR | 95%CI | 0.87 (0.61 to 1.23) | NR | NR | NR | EV < 1 |
| 39 | Trial protocol registered | REG (NO vs YES) | NR | NR | NR | ROR | 95%CI | 1.30 (0.83 to 2.01) | NR | NR | NR | EV < 1 |
| 40 | Multicenter study | MC (NO vs YES) | NR | NR | NR | ROR | 95%CI | 0.96 (0.68 to 1.38) | NR | NR | NR | EV < 1 |
| 41 | Industry funded | IND (YES vs NO) | NR | NR | NR | ROR | 95%CI | 0.79 (0.54 to 1.16) | NR | NR | NR | EV < 1 |
| 42 | Trialists with conflict of interest | COI (YES vs No) | NR | NR | NR | ROR | 95%CI | 0.72 (0.49 to 1.06) | NR | NR | NR | EV < 1 |
| 43 | Random allocation | RS (IN/UN vs A) | NR | NR | NR | ROR | 95%CI | 0.98 (0.73 to 1.31) | NR | NR | NR | EV < 1 |
| 44 | Allocation concealment | AC (IN/UN vs A) | NR | NR | NR | ROR | 95%CI | 1.13 (0.70 to 1.81) | NR | NR | NR | EV < 1 |
| 45 | Observer blinding | BO (NO/UN vs Yes) | NR | NR | NR | ROR | 95%CI | 0.81 (0.48 to 1.37) | NR | NR | NR | EV < 1 |
| 46 | Patients blinding | BP (NO vs Yes) | NR | 18 | NR | ROR | 95%CrI | 0.91 (0.61 to 1.34) | NR | NR | NR | EV < 1 |
| 47 | Observer blinding | BO (NO vs Yes) | NR | 14 | NR | ROR | 95%CrI | 0.98 (0.69 to 1.39) | NR | NR | NR | EV < 1 |
| 48 | Personnel blinding | BS (NO vs Yes) | NR | 29 | NR | ROR | 95%CrI | 1.01 (0.84 to 1.19) | NR | NR | NR | EV < 1 |
| 49 | Double blinding | DB (NO vs Yes) | NR | 74 | NR | ROR | 95%CrI | 1.02 (0.90 to 1.13) | ϕ | 95%CrI | 0.06 (0.01 to 0.27) | EV < 1 |
| 50 | Patients blinding | BP (NO vs Yes) | NR | NR | 4 | ES | 95%CI | -0.19 (-0.48 to 0.10) | I^2^ | NR | 91.4% | EV < 0 |
| 51 | Observer blinding | BO (NO vs Yes) | NR | NR | 4 | ES | 95%CI | -0.10 (-0.31 to 0.11) | I^2^ | NR | 71.9% | EV < 0 |
| 52 | Random allocation | RS (NO vs YES) | NR | NR | 160 | ROR | 95%CI | 1.04 (0.88 to 1.23) | I^2^ | 95%CI | 3% (0 to 56%) | EV < 1 |
| 53 | Trial protocol registered | REG (NO vs YES) | NR | NR | NR | Log OR | 95%CI | 0.03 (-0.03 to 0.09) | I^2^ | NR | 1% | EV < 0 |
| 54 | Industry funded | IND (YES vs NO) | NR | NR | 363 | sROR | 95%CI | 1.10 (0.96 to 1.26) | I^2^ | NR | 1% (0 to 40%) | EV < 1 |
| 55 | Trial protocol registered | REG (NO vs YES) | NR | 16 | 83 | dSMD | 95%CI | -0.36 (-0.60 to –0.12) | I^2^ | 95%CI | 0% (0 to 42%) | EV < 0 |
| 56 | Random allocation | RS (H/UN vs L) | NR | 189 | 2158 | ROR | 95%CrI | 0.91 (0.86 to 0.98) | ϕ | 95%CrI | 0.10 (0.02 to 0.20) | EV < 1 |
| 57 | Allocation concealment | AC (H/UN vs L) | NR | 188 | 2121 | ROR | 95%CrI | 0.92 (0.86 to 0.98) | ϕ | 95%CrI | 0.05 (0.01 to 0.17) | EV < 1 |
| 58 | Blinding | BLI (H/UN vs L) | NR | 144 | 1678 | ROR | 95%CrI | 0.87 (0.80 to 0.93) | ϕ | 95%CrI | 0.12 (0.02 to 0.24) | EV < 1 |
| 59 | Incomplete outcome data control | IDC (H/UN vs L) | NR | 167 | 1956 | ROR | 95%CrI | 0.98 (0.92 to 1.05) | ϕ | 95%CrI | 0.05 (0.01 to 0.15) | EV < 1 |
| 60 | Random allocation | RS (IN/UN vs A) | NR | 52 | 467 | ES | 95%CI | 0.13 (0.01 to 0.25) | I^2^ | NR | 44.7% | EV < 0 |
| 61 | Allocation concealment | AC (IN/UN vs A) | NR | 39 | 345 | ES | 95%CI | 0.15 (0.02 to 0.27) | NR | NR | NR | EV < 0 |
| 62 | Baseline imbalance | BSL (YES vs NO) | NR | 32 | 310 | ES | 95%CI | 0.01 (–0.09 to 0.12) | NR | NR | NR | EV < 0 |
| 63 | Trial protocol registered | REG (UR/RR vs PR) | NR | 32 | 165 | ROR | 95%CI | 0.81 (0.65 to 1.02) | I^2^ | NR | 21.6% | EV < 1 |
| 64 | Trial protocol registered | REG (NO vs YES) | NR | 37 | 177 | ROR | 95%CI | 0.85 (0.67 to 1.08) | I^2^ | NR | 37.2% | EV < 1 |
| 65 | Trial protocol registered | REG (PAD/NO vs PCD | NR | 43 | 213 | ROR | 95%CI | 0.84 (0.71 to 1.01) | I^2^ | NR | 17.5% | EV < 1 |
| 66 | Patients blinding | BP (IN vs A) | NR | 9 | 121 | ES | 95%CI | -0.12 (-0.30 to 0.06) | I^2^ | NR | 0% | EV < 0 |
| 67 | Observer blinding | BO (IN vs A) | NR | 17 | 165 | ES | 95%CI | -0.07 (-0.22 to 0.08) | I^2^ | NR | 10.2% | EV < 0 |
| 68 | Personnel blinding | BS (IN vs A) | NR | 7 | 113 | ES | 95%CI | 0.10 (-0.17 to 0.38) | NR | NR | NR | EV < 0 |
| 69 | Statistician blinding | BST (IN vs A) | NR | 6 | 89 | ES | 95%CI | 0.04 (-0.18 to 0.27) | NR | NR | NR | EV < 0 |
| 70 | Blinding | BLI (IN vs A) | NR | 16 | 182 | ES | 95%CI | -0.08 (-0.28 to 0.12) | NR | NR | NR | EV < 0 |
| 71 | Use of historic (untreated) control group | HIST (YES vs NO) | NR | 13 | 171 | dSMD | 95%CI | -0.31 (-0.53 to –0.10) | I^2^ | 95%CI | 0% (0 to 49%) | EV < 0 |
| 72 | Random allocation | RS (NO vs YES) | NR | 25 | NR | dSMD | 95%CI | 0.07 (-0.21 to 0.34) | NR | NR | NR | EV < 0 |
| 73 | Prospective trials | PRO (RET vs YES) | NR | 40 | NR | dSMD | 95%CI | -0.30 (-0.53 to –0.06) | NR | NR | NR | EV < 0 |
| 74 | Random allocation | RS (IN/UN vs A) | NR | 25 | NR | dSMD | 95%CI | 0.01 (-0.25 to 0.26) | NR | NR | NR | EV < 0 |
| 75 | Multicenter study | MC (NO vs YES) | NR | 48 | 421 | ROR | 95%CI | 0.73 (0.64 to 0.83) | I^2^ | NR | 12% | EV < 1 |
| 76 | Sample size | SS3 (NO vs YES) | NR | 52 | 417 | ROR | 95%CI | 0.56 (0.45 to 0.70) | τ^2^ | NR | 0 | EV < 1 |
| 77 | Sample size | SS1 (NO vs YES) | NR | 72 | 602 | ROR | 95%CI | 0.74 (0.65 to 0.85) | τ^2^ | NR | 0 | EV < 1 |
| 78 | Sample size | SS2 (NO vs YES) | NR | 67 | 598 | ROR | 95%CI | 0.76 (0.68 to 0.85) | τ^2^ | NR | 0.02 | EV < 1 |
| 79 | Sample size | SS4 (NO vs YES) | NR | 43 | 434 | ROR | 95%CI | 0.81 (0.74 to 0.89) | τ^2^ | NR | 0 | EV < 1 |
| 80 | Sample size | SS5 (NO vs YES) | NR | 28 | 301 | ROR | 95%CI | 0.82 (0.76 to 0.90) | τ^2^ | NR | 0 | EV < 1 |
| 81 | Sample size | SS1 (NO vs YES) | NR | 13 | 153 | ES | 95%CI | -0.21 (-0.34 to –0.08) | τ^2^ | NR | 0.03 | EV < 0 |
| 82 | Allocation concealment | AC (UN vs A) | NR | NR | 26 | ES | 95%CI | 0.22 (-0.58 to 1.03) | NR | NR | NR | EV < 0 |
| 83 | Allocation concealment | AC (IN vs A) | NR | NR | 10 | ES | 95%CI | 0.60 (-1.70 to 1.89) | NR | NR | NR | EV < 0 |
| 84 | Allocation concealment | AC (IN/UN vs A) | NR | NR | 34 | ES | 95%CI | 0.25 (-0.53 to 1.03) | NR | NR | NR | EV < 0 |
| 85 | Observer blinding | BO (IN vs A) | NR | NR | 33 | ES | 95%CI | -0.20 (-0.76 to 0.36) | NR | NR | NR | EV < 0 |
| 86 | Random allocation | RS (IN vs A) | NR | 12 | 165 | ESD | 95%CI | 0.01 (-0.15 to 0.17) | NR | NR | NR | EV > 0 |
| 87 | Allocation concealment | AC (IN vs A) | NR | 12 | 165 | ESD | 95%CI | -0.05 (-0.22 to 0.11) | NR | NR | NR | EV > 0 |
| 88 | Baseline imbalance | BSL (IN vs A) | NR | 12 | 165 | ESD | 95%CI | -0.09 ( -0.24 to 0.05) | NR | NR | NR | EV > 0 |
| 89 | Observer blinding | BO (IN vs A) | NR | 12 | 165 | ESD | 95%CI | 0.06 (-0.28 to 0.41) | NR | NR | NR | EV > 0 |
| 90 | Personnel blinding | BS (IN vs A) | NR | 12 | 165 | ESD | 95%CI | 0.19 (0.03 to 0.35) | NR | NR | NR | EV > 0 |
| 91 | Patients blinding | BP (IN vs A) | NR | 12 | 165 | ESD | 95%CI | 0.21 (0.04 to 0.39) | NR | NR | NR | EV > 0 |
| 92 | Intention-to-treat analysis | ITT (IN vs A) | NR | 12 | 165 | ESD | 95%CI | 0.05 (-0.10 to 0.20) | NR | NR | NR | EV > 0 |
| 93 | Crossover design | CRO (YES vs PR) | NR | NR | 34 | OE | 95%CI | 60% (16 to 119%) | NR | NR | NR | EV > 0 |
| 94 | Allocation concealment | AC (IN vs A) | NR | NR | 34 | OE | 95%CI | 57% (2 to 140%) | NR | NR | NR | EV > 0 |
| 95 | Blinding | BLI (IN vs A) | NR | NR | 34 | OE | 95%CI | 41% (2 to 94%) | NR | NR | NR | EV > 0 |
| 96 | Follow-up | FO (IN vs A) | NR | NR | 34 | UE | 95%CI | 25% (3 to 42%) | NR | NR | NR | EV > 0 |
| 97 | Crossover design | CRO (YES vs PR) | NR | NR | 26 | ROR | 95%CI | 0.87 (0.74 to 1.02) | I^2^ | NR | 0% | EV < 1 |
| 98 | Allocation concealment | AC (IN vs A) | NR | NR | 429 | RHR | 95%CI | 0.94 (0.88 to 0.99) | NR | NR | NR | EV < 1 |
| 99 | Blinding | BLI (IN vs A) | NR | NR | 429 | RHR | 95%CI | 1.24 (1.05 to 1.43) | NR | NR | NR | EV < 1 |
| 100 | Allocation concealment | AC (IN vs A) | NR | 11 | 127 | ROR | 95%CI | 0.63 (0.45 to 0.88) | 2p-values | NR | 0.7135 | EV < 1 |
| 103 | Double blinding | DB (NO/UN vs YES) | NR | 112 | 1057 | ROR | 95%CrI | 0.87 (0.79 to 0.96) | SD increase | 95%CrI | 0.14 (0.02 to 0.30) | EV < 1 |
| 104 | Random allocation | RS (IN/UN vs A) | NR | 146 | 1292 | ROR | 95%CrI | 0.89 (0.82 to 0.96) | SD increase | 95%CrI | 0.16 (0.03 to 0.27) | EV < 1 |
| 105 | Allocation concealment | AC (IN/UN vs A) | NR | 104 | 1057 | ROR | 95%CrI | 0.93 (0.87 to 0.99) | SD increase | 95%CrI | 0.12 (0.02 to 0.23) | EV < 1 |
| 106 | Sample size | SS (small vs large) | NR | 16 | 229 | ROR | 95%CI | 1.32 (0.89 to 1.75) | I^2^ | 95%CI | 75.5% | EV < 1 |

ME = Meta-epidemiological studies; MA’s = Meta-analyses; I^2^ = Reported in-between meta-analysis or trial heterogeneity (%); dSMD = Difference in standardized mean differences; ES = Effect size estimate; ESD = Effect size difference; CI = Confidence interval; CrI = Credible interval; RC = Regression coefficient; OR = Odds ratio; sROR = Summary ROR across topics; ϕ = Between-MA Heterogeneity; OE = Overestimation; UE = Underestimation; RHR = Ratio of Hazard Ratios;

**Excluded datasets**

Total number = 77

| Reason for dataset exclusion: Low confidence for low Quality of evidence (QoE) = 5 | | | | | | | | | | | | | | |
| --- | --- | --- | --- | --- | --- | --- | --- | --- | --- | --- | --- | --- | --- | --- |
| Study ID | | Reference | | | | | | | | | | | | Dataset ID |
| 44 | | Armijo-Olivo S, da Costa BR, Ha C, Saltaji H, Cummings GG, Fuentes J. Are biases related to attrition, missing data, and the use of intention to treat related to the magnitude of treatment effects in physical therapy trials? A meta-epidemiological study. Am J Phys Med Rehabil. 2021 Jul 3. doi: 10.1097/PHM.0000000000001837. | | | | | | | | | | | | 25 |
| 60 | | Savovic J, Turner RM, Mawdsley D, Jones HE, Beynon R, Higgins JPT, Sterne JAC. Association Between Risk-of-Bias Assessments and Results of Randomized Trials in Cochrane Reviews: The ROBES Meta-Epidemiologic Study. Am J Epidemiol. 2018 May 1;187(5):1113-1122. | | | | | | | | | | | | 56 |
|  |  |  |  |  |  |  |  |  |  |  |  |  |  | 57 |
|  |  |  |  |  |  |  |  |  |  |  |  |  |  | 58 |
| 79 | | Khan KS, Daya S, Collins JA, Walter SD. Empirical evidence of bias in infertility research: overestimation of treatment effect in crossover trials using pregnancy as the outcome measure. Fertil Steril. 1996 May;65(5):939-45. | | | | | | | | | | | | 96 |
| **Reason for dataset exclusion: Datasets with statistically significant result for a trial design characteristic for which also datasets with non-significant results were found but which cannot be pooled = 15** | | | | | | | | | | | | | | |
| 84 | | Page MJ, Higgins JP, Clayton G, Sterne JA, Hróbjartsson A, Savović J. Empirical Evidence of Study Design Biases in Randomized Trials: Systematic Review of Meta-Epidemiological Studies. PLoS One. 2016 Jul 11;11(7):e0159267. | | | | | | | | | | | | 1 |
|  |  |  |  |  |  |  |  |  |  |  |  |  |  | 2 |
| 44 | | Armijo-Olivo S, da Costa BR, Ha C, Saltaji H, Cummings GG, Fuentes J. Are biases related to attrition, missing data, and the use of intention to treat related to the magnitude of treatment effects in physical therapy trials? A meta-epidemiological study. Am J Phys Med Rehabil. 2021 Jul 3. doi: 10.1097/PHM.0000000000001837. | | | | | | | | | | | | 27 |
| 61 | | Saltaji H, Armijo-Olivo S, Cummings GG, Amin M, da Costa BR, Flores-Mir C. Impact of Selection Bias on Treatment Effect Size Estimates in Randomized Trials of Oral Health Interventions: A Meta-epidemiological Study. J Dent Res. 2018 Jan;97(1):5-13. | | | | | | | | | | | | 60 |
| 72 | | Dechartres A, Trinquart L, Boutron I, Ravaud P. Influence of trial sample size on treatment effect estimates: meta-epidemiological study. BMJ. 2013 Apr 24;346:f2304. | | | | | | | | | | | | 76 |
| 76 | | Hempel S, Miles J, Suttorp MJ, Wang Z, Johnsen B, Morton S, Perry T, Valentine D, Shekelle PG. Detection of Associations Between Trial Quality and Effect Sizes [Internet]. Rockville (MD): Agency for Healthcare Research and Quality (US); 2012 Jan. Report No.: 12-EHC010-EF.  . | | | | | | | | | | | | 90 |
|  |  |  |  |  |  |  |  |  |  |  |  |  |  | 91 |
| 79 | | Khan KS, Daya S, Collins JA, Walter SD. Empirical evidence of bias in infertility research: overestimation of treatment effect in crossover trials using pregnancy as the outcome measure. Fertil Steril. 1996 May;65(5):939-45. | | | | | | | | | | | | 93 |
|  |  |  |  |  |  |  |  |  |  |  |  |  |  | 94 |
|  |  |  |  |  |  |  |  |  |  |  |  |  |  | 95 |
| 81 | | Mhaskar R, Djulbegovic B, Magazin A, Soares HP, Kumar A. Published methodological quality of randomized controlled trials does not reflect the actual quality assessed in protocols. J Clin Epidemiol. 2012 Jun;65(6):602-9. | | | | | | | | | | | | 98 |
|  |  |  |  |  |  |  |  |  |  |  |  |  |  | 99 |
| 82 | | Moher D, Cook DJ, Jadad AR, Tugwell P, Moher M, Jones A, Pham B, Klassen TP. Assessing the quality of reports of randomised trials: implications for the conduct of meta-analyses. Health Technol Assess. 1999;3(12):i-iv, 1-98. | | | | | | | | | | | | 100 |
| 74 | | Savović J, Jones HE, Altman DG, Harris RJ, Jüni P, Pildal J, Als-Nielsen B, Balk EM, Gluud C, Gluud LL, Ioannidis JP, Schulz KF, Beynon R, Welton NJ, Wood L, Moher D, Deeks JJ, Sterne JA. Influence of reported study design characteristics on intervention effect estimates from randomized, controlled trials. Ann Intern Med. 2012 Sep 18;157(6):429-38. | | | | | | | | | | | | 104 |
|  |  |  |  |  |  |  |  |  |  |  |  |  |  | 105 |
| Reason for dataset exclusion: Not relevant for prospective, clinical controlled trials = 2 | | | | | | | | | | | | | | |
| 64 | | Papageorgiou SN, Koretsi V, Jäger A. Bias from historical control groups used in orthodontic research: a meta-epidemiological study. Eur J Orthod. 2017 Feb;39(1):98-105. | | | | | | | | | | | | 71 |
| 68 | | Papageorgiou SN, Xavier GM, Cobourne MT. Basic study design influences the results of orthodontic clinical investigations. J Clin Epidemiol. 2015 Dec;68(12):1512-22. | | | | | | | | | | | | 73 |
| Reason for dataset exclusion: Duplication of reported data = 1 | | | | | | | | | | | | | | |
| 84 | | Page MJ, Higgins JP, Clayton G, Sterne JA, Hróbjartsson A, Savović J. Empirical Evidence of Study Design Biases in Randomized Trials: Systematic Review of Meta-Epidemiological Studies. PLoS One. 2016 Jul 11;11(7):e0159267. (same as Study ID 74) | | | | | | | | | | | | 11 |
| **Reason for dataset exclusion: No statistically significant effect estimate established = 54** | | | | | | | | | | | | | | |
| Dataset ID | Trial design characteristic investigated | | Comparison type | Number of included | | | Type of estimate | Type of estimate precision measure | Estimate value (EV) | Type of heterogeneity estimate | Type of heterogeneity precision measure | Heterogeneity value | Direction of effect for over-estimation | |
|  |  |  |  | ME | MA | Trials |  |  |  |  |  |  |  |  |
| 03 | Baseline imbalance | | BSL (YES vs No) | 24 | NR | NR | ROR | 95%CI | 1.03 (0.89 to 1.19) | I^2^ | NR | 0% | EV < 1 | |
| 04 | Confounder adjustment | | CO (NO vs YES) | 24 | NR | NR | ROR | 95%CI | 0.96 (0.79 to 1.23) | NR | NR | NR | EV < 1 | |
| 05 | Block randomisation | | BR (NO vs YES) | 24 | NR | NR | dSMD | 95%CI | -0.18 (-0.47 to 0.11) | NR | NR | NR | EV < 0 | |
| 06 | Patients blinding | | BP (NO/UN vs Yes) | 24 | NR | NR | ROR | 95%CI | 0.92 (0.81 to 1.04) | I^2^ | NR | 0% | EV < 1 | |
| 07 | Personnel blinding | | BS (NO/UN vs Yes) | 24 | NR | NR | ROR | 95%CI | 1.00 (0.86 to 1.16) | I^2^ | NR | 0% | EV < 1 | |
| 09 | Drop-out rate | | LTF (YES vs No) | 24 | NR | NR | ROR | 95%CI | 1.07 (0.92 to 1.25) | NR | NR | NR | EV < 1 | |
| 10 | Observer blinding | | BO (NO/UN vs Yes) | 24 | NR | NR | ROR | 95%CI | 1.01 (0.90 to 1.13) | I^2^ | NR | 0% | EV < 1 | |
| 12 | Selective outcome reporting | | SR (YES/UN vs A} | 24 | NR | NR | ROR | 95%CI | 0.71 (0.43 to 1.19) | NR | NR | NR | EV < 1 | |
| 13 | Intention-to-treat analysis | | ITT (NO vs DR) | NR | 50 | 226 | ROR | 95%CI | 0.92 (0.70 to 1.23) | τ^2^ | NR | 0.57 | EV < 1 | |
| 14 | Random allocation | | RS (IN vs A) | NR | 43 | 393 | ES | 95%CI | 0.02 (-0.12 to 0.15) | NR | NR | NR | EV < 0 | |
| 20 | One randomised arm | | RL (YES vs NO) | NR | 36 | 443 | ROR | 95%CI | 0.99 (0.91 to 1.08) | I^2^ | 95%CI | 0% (0 to 34%) | EV < 1 | |
| 26 | Intention-to-treat analysis | | ITT (N/UN vs YES) | NR | 37 | 359 | ES | 95%CI | -0.04 (-0.14 to 0.06) | NR | NR | NR | EV > 0 | |
| 28 | Random allocation | | RS (IN/UN vs A) | NR | NR | NR | ROR | 95%CI | 1.04 (0.93 to 1.16) | NR | NR | NR | EV < 1 | |
| 29 | Allocation concealment | | AC (IN/UN vs A) | NR | NR | NR | ROR | 95%CI | 1.10 (0.93 to 1.16) | NR | NR | NR | EV < 1 | |
| 30 | Observer blinding | | BO (NO/UN vs Yes) | NR | NR | NR | ROR | 95%CI | 0.97 (0.86 to 1.08) | NR | NR | NR | EV < 1 | |
| 31 | Incomplete outcome data control | | IDC (IN/UN vs A) | NR | NR | NR | ROR | 95%CI | 1.11 (0.99 to 1.24) | NR | NR | NR | EV < 1 | |
| 32 | Multicenter study | | MC (NO vs YES) | NR | NR | NR | ROR | 95%CI | 1.04 (0.93 to 1.16) | NR | NR | NR | EV < 1 | |
| 33 | Trial stopped early | | TS (YES/UN vs NO) | NR | NR | NR | ROR | 95%CI | 0.93 (0.78 to 1.12) | NR | NR | NR | EV < 1 | |
| 34 | For-profit funding source | | FPF (YES vs NO) | NR | NR | NR | ROR | 95%CI | 0.97 (0.85 to 1.11) | NR | NR | NR | EV < 1 | |
| 38 | Sample size | | SS3 (NO vs YES) | NR | NR | NR | ROR | 95%CI | 0.87 (0.61 to 1.23) | NR | NR | NR | EV < 1 | |
| 39 | Trial protocol registered | | REG (NO vs YES) | NR | NR | NR | ROR | 95%CI | 1.30 (0.83 to 2.01) | NR | NR | NR | EV < 1 | |
| 40 | Multicenter study | | MC (NO vs YES) | NR | NR | NR | ROR | 95%CI | 0.96 (0.68 to 1.38) | NR | NR | NR | EV < 1 | |
| 41 | Industry funded | | IND (YES vs NO) | NR | NR | NR | ROR | 95%CI | 0.79 (0.54 to 1.16) | NR | NR | NR | EV < 1 | |
| 42 | Trialists with conflict of interest | | COI (YES vs No) | NR | NR | NR | ROR | 95%CI | 0.72 (0.49 to 1.06) | NR | NR | NR | EV < 1 | |
| 43 | Random allocation | | RS (IN/UN vs A) | NR | NR | NR | ROR | 95%CI | 0.98 (0.73 to 1.31) | NR | NR | NR | EV < 1 | |
| 44 | Allocation concealment | | AC (IN/UN vs A) | NR | NR | NR | ROR | 95%CI | 1.13 (0.70 to 1.81) | NR | NR | NR | EV < 1 | |
| 45 | Observer blinding | | BO (NO/UN vs Yes) | NR | NR | NR | ROR | 95%CI | 0.81 (0.48 to 1.37) | NR | NR | NR | EV < 1 | |
| 46 | Patients blinding | | BP (NO vs Yes) | NR | 18 | NR | ROR | 95%CrI | 0.91 (0.61 to 1.34) | NR | NR | NR | EV < 1 | |
| 47 | Observer blinding | | BO (NO vs Yes) | NR | 14 | NR | ROR | 95%CrI | 0.98 (0.69 to 1.39) | NR | NR | NR | EV < 1 | |
| 48 | Personnel blinding | | BS (NO vs Yes) | NR | 29 | NR | ROR | 95%CrI | 1.01 (0.84 to 1.19) | NR | NR | NR | EV < 1 | |
| 49 | Double blinding | | DB (NO vs Yes) | NR | 74 | NR | ROR | 95%CrI | 1.02 (0.90 to 1.13) | NR | NR | NR | EV < 1 | |
| 50 | Patients blinding | | BP (NO vs Yes) | NR | NR | 4 | ES | 95%CI | -0.19 (-0.48 to 0.10) | I^2^ | NR | 91.4% | EV < 0 | |
| 51 | Observer blinding | | BO (NO vs Yes) | NR | NR | 4 | ES | 95%CI | -0.10 (-0.31 to 0.11) | I^2^ | NR | 71.9% | EV < 0 | |
| 52 | Random allocation | | RS (NO vs YES) | NR | NR | 160 | ROR | 95%CI | 1.04 (0.88 to 1.23) | I^2^ | 95%CI | 3% (0 to 56%) | EV < 1 | |
| 53 | Trial protocol registered | | REG (NO vs YES) | NR | NR | NR | Log OR | 95%CI | 0.03 (-0.03 to 0.09) | I^2^ | NR | 1% | EV < 0 | |
| 54 | Industry funded | | IND (YES vs NO) | NR | NR | 363 | sROR | 95%CI | 1.10 (0.96 to 1.26) | I^2^ | NR | 1% (0 to 40%) | EV < 1 | |
| 59 | Incomplete outcome data control | | IDC (H/UN vs L) | NR | 167 | 1956 | ROR | 95%CrI | 0.98 (0.92 to 1.05) | ϕ | 95%CrI | 0.05 (0.01 to 0.15) | EV < 1 | |
| 62 | Baseline imbalance | | BSL (YES vs NO) | NR | 32 | 310 | ES | 95%CI | 0.01 (–0.09 to 0.12) | NR | NR | NR | EV < 0 | |
| 63 | Trial protocol registered | | REG (UR/RR vs PR) | NR | 32 | 165 | ROR | 95%CI | 0.81 (0.65 to 1.02) | I^2^ | NR | 21.6% | EV < 1 | |
| 65 | Trial protocol registered | | REG (PAD/NO vs PCD | NR | 43 | 213 | ROR | 95%CI | 0.84 (0.71 to 1.01) | I^2^ | NR | 17.5% | EV < 1 | |
| 66 | Patients blinding | | BP (IN vs A) | NR | 9 | 121 | ES | 95%CI | -0.12 (-0.30 to 0.06) | I^2^ | NR | 0% | EV < 0 | |
| 68 | Personnel blinding | | BS (IN vs A) | NR | 7 | 113 | ES | 95%CI | 0.10 (-0.17 to 0.38) | NR | NR | NR | EV < 0 | |
| 69 | Statistician blinding | | BST (IN vs A) | NR | 6 | 89 | ES | 95%CI | 0.04 (-0.18 to 0.27) | NR | NR | NR | EV < 0 | |
| 70 | Blinding | | BLI (IN vs A) | NR | 16 | 182 | ES | 95%CI | -0.08 (-0.28 to 0.12) | NR | NR | NR | EV < 0 | |
| 72 | Random allocation | | RS (NO vs YES) | NR | 25 | NR | dSMD | 95%CI | 0.07 (-0.21 to 0.34) | NR | NR | NR | EV < 0 | |
| 74 | Random allocation | | RS (IN/UN vs A) | NR | 25 | NR | dSMD | 95%CI | 0.01 (-0.25 to 0.26) | NR | NR | NR | EV < 0 | |
| 82 | Allocation concealment | | AC (UN vs A) | NR | NR | 26 | ES | 95%CI | 0.22 (-0.58 to 1.03) | NR | NR | NR | EV < 0 | |
| 86 | Random allocation | | RS (IN vs A) | NR | 12 | 165 | ESD | 95%CI | 0.01 (-0.15 to 0.17) | NR | NR | NR | EV > 0 | |
| 87 | Allocation concealment | | AC (IN vs A) | NR | 12 | 165 | ESD | 95%CI | -0.05 (-0.22 to 0.11) | NR | NR | NR | EV > 0 | |
| 88 | Baseline imbalance | | BSL (IN vs A) | NR | 12 | 165 | ESD | 95%CI | -0.09 ( -0.24 to 0.05) | NR | NR | NR | EV > 0 | |
| 89 | Observer blinding | | BO (IN vs A) | NR | 12 | 165 | ESD | 95%CI | 0.06 (-0.28 to 0.41) | NR | NR | NR | EV > 0 | |
| 92 | Intention-to-treat analysis | | ITT (IN vs A) | NR | 12 | 165 | ESD | 95%CI | 0.05 (-0.10 to 0.20) | NR | NR | NR | EV > 0 | |
| 106 | Sample size | | SS (small vs large) | NR | 16 | 229 | ROR | 95%CI | 1.32 (0.89 to 1.75) | I^2^ | 95%CI | 75.5% | EV < 1 | |
| 35 | Intention-to-treat analysis | | ITT (PP vs YES) | NR | NR | 156 | ROR | 95%CI | 1.02 (1.00 to 1.04)* | NR | NR | NR | EV > 1 | |

*p = 0.03 but statistically borderline significance, only, with LCL = 1.00 value.

**Datasets for meta-analysis**

Total number of datasets = 21

Total number of possible meta-analyses (MA) = 10 / Excluded MA = 2 / Included MA = 8

| MA-ID | Study ID | DS-ID | Comparison type | Trials(N) | TO | Type of Estimate | EV | Type of precision measure | LCL | UCL | Direction of effect for over-estimation | MA | Reason for exclusion |
| --- | --- | --- | --- | --- | --- | --- | --- | --- | --- | --- | --- | --- | --- |
| 01 | 12 | 15 | AC (IN vs A) | 393 | 1 | ES | 0.12 | 95%CI | -0.06 | 0.3 | EV < 0 | Included | - |
|  | 75 | 83 | AC (IN vs A) | 10 | 1 | ES | 0.6 | 95%CI | -1.7 | 1.89 | EV < 0 |  |  |
| 02 | 61 | 61 | AC (IN/UN vs A) | 345 | 1 | ES | 0.15 | 95%CI | 0.02 | 0.27 | EV < 0 | Included | - |
|  | 75 | 84 | AC (IN/UN vs A) | 34 | 1 | ES | 0.25 | 95%CI | -0.53 | 1.03 | EV < 0 |  |  |
| 03 | 63 | 67 | BO (IN vs A) | 165 | 1 | ES | -0.07 | 95%CI | -0.22 | 0.08 | EV < 0 | Included | - |
|  | 75 | 85 | BO (IN vs A) | 33 | 1 | ES | -0.2 | 95%CI | -0.76 | 0.36 | EV < 0 |  |  |
| 04 | 26 | 16 | MC (NO vs YES) | 292 | 2 | dSMD | -0.09 | 95%CI | -0.17 | -0.01 | EV < 0 | Included | - |
|  | 71 | 75 | MC (NO vs YES) | 421 | 1 | ROR | 0.73 | 95%CI | 0.64 | 0.83 | EV < 1 |  |  |
| 05 | 41 | 21 | PA (NO vs YES) | 56 | 1 | ROR | 0.96 | 95%CI | 0.52 | 1.8 | EV < 1 | Excluded | Not relevant for prospective, clinical controlled trials |
|  | 41 | 22 | PA (NO vs YES) | 56 | 2 | dSMD | 0.08 | 95%CI | -0.14 | 0.3 | EV < 0 |  |  |
| 06 | 59 | 55 | REG (NO vs YES) | 83 | 2 | dSMD | -0.36 | 95%CI | -0.6 | -0.12 | EV < 0 | Included | - |
|  | 62 | 64 | REG (NO vs YES) | 177 | 1 | ROR | 0.85 | 95%CI | 0.67 | 1.08 | EV < 1 |  |  |
| 07 | 29 | 17 | RS (NO vs YES) | 164 | 1 | ROR | 0.83 | 95%CI | 0.65 | 1.04 | EV < 1 | Included | - |
|  | 35 | 19 | RS (NO vs YES) | 246 | 1 | ROR | 0.94 | 95%CI | 0.77 | 1.16 | EV < 1 |  |  |
|  | 54 | 52 | RS (NO vs YES) | 160 | 1 | ROR | 1.04 | 95%CI | 0.88 | 1.23 | EV < 1 |  |  |
| 08 | 43 | 24 | SPO (H/UN vs L) | 540 | 1 | ES | 0.1 | 95%CI | 0.02 | 0.19 | EV > 0 | Excluded | Low confidence for low Quality of evidence (QoE) |
|  | 49 | 37 | SPO (H/UN vs L) | 393 | 1 | ES | 0.15 | 95%CI | -0.03 | 0.33 | EV > 0 |  |  |
| 09 | 42 | 23 | SS1 (NO vs YES) | 317 | 1 | ROR | 0.6 | 95%CI | 0.53 | 0.68 | EV < 1 | Included | - |
|  | 72 | 77 | SS1 (NO vs YES) | 602 | 1 | ROR | 0.74 | 95%CI | 0.65 | 0.85 | EV < 1 |  |  |
| 10 | 35 | 18 | SS2 (NO vs YES) | 619 | 1 | ROR | 0.92 | 95%CI | 0.87 | 0.98 | EV < 1 | Included | - |
|  | 72 | 78 | SS2 (NO vs YES) | 598 | 1 | ROR | 0.76 | 95%CI | 0.68 | 0.85 | EV < 1 |  |  |

**Meta-analysis results**

MA-ID 01 (Effect estimate: dSMD)


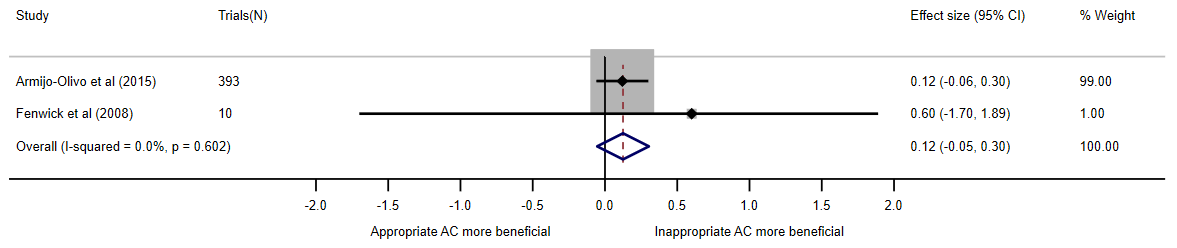


MA-ID 02 (Effect estimate: dSMD)

**
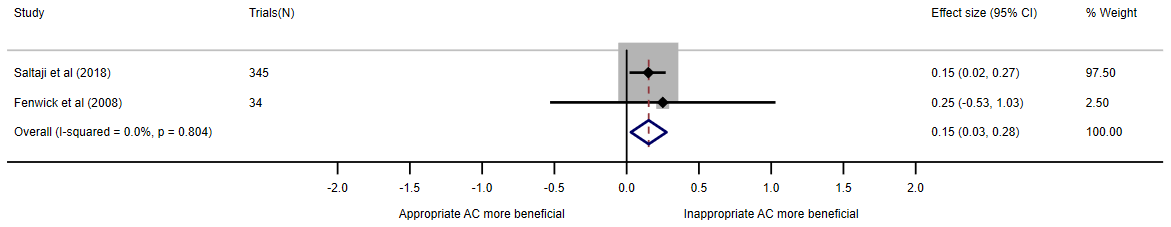
**

MA-ID 03 (Effect estimate: dSMD)

**
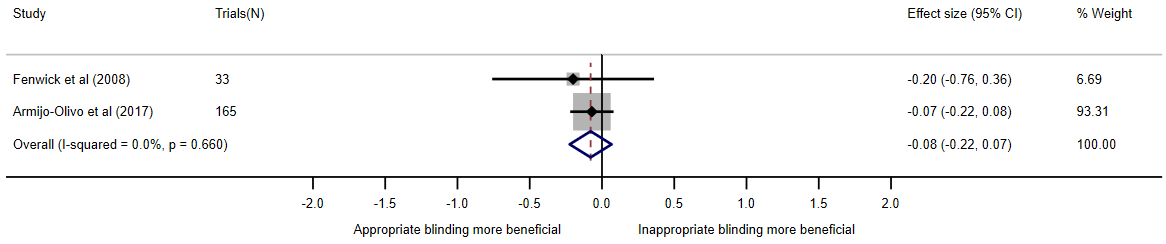
**

MA-ID 04 (Effect estimate: ROR)

**
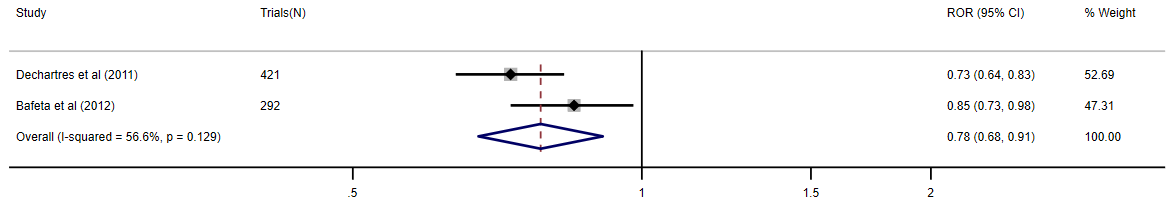
**

MA-ID 06 (Effect estimate: ROR)

**
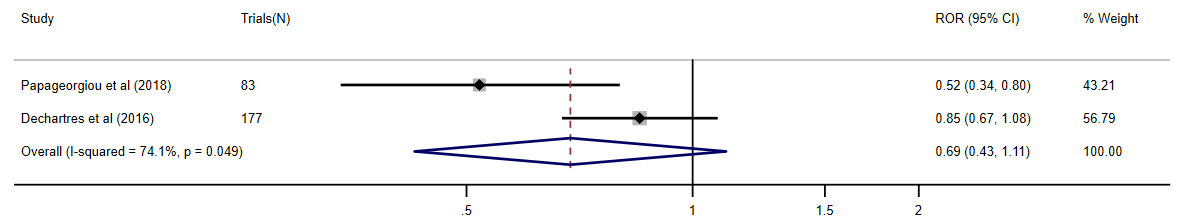
**

MA-ID 07 (Effect estimate: ROR)

**
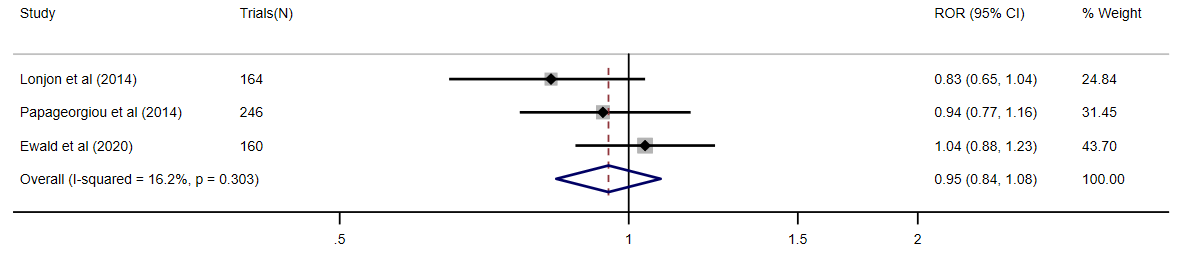
**

MA-ID 09 (Effect estimate: ROR)


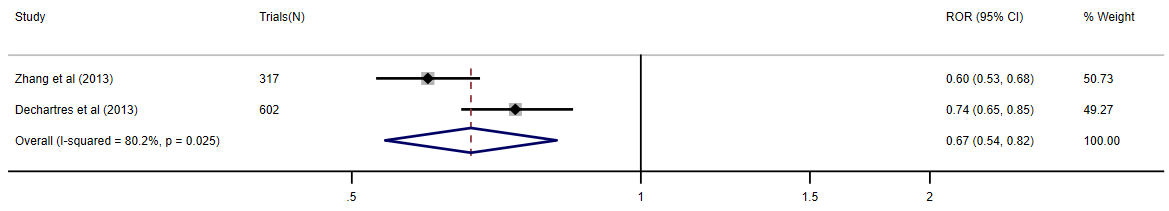


MA-ID 10 (Effect estimate: ROR)

**
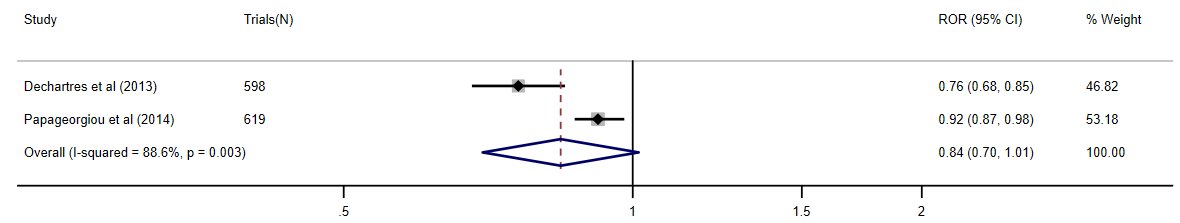
**

**Included single datasets**

Total number = 6

| DS-ID | Study-ID | Comparison type | Trials(N) | Type of Estimate | EV | Type of precision measure | LCL | UCL | Direction of effect for over-estimation |
| --- | --- | --- | --- | --- | --- | --- | --- | --- | --- |
| 36 | 47 | DB (NO/UN vs YES) | 467 | ROR | 0.91 | 95%CI | 0.84 | 0.99 | EV < 1 |
| 103 | 74 | DB (NO/UN vs YES) | 1057 | ROR | 0.87 | 95%CrI | 0.79 | 0.96 | EV < 1 |
| 08 | 11 | ITT (DR vs YES) | 202 | ROR | 0.8 | 95%CI | 0.69 | 0.94 | EV < 1 |
| 81 | 73 | SS1 (NO vs YES) | 153 | ES | -0.21 | 95%CI | -0.34 | -0.08 | EV < 0 |
| 79 | 72 | SS4 (NO vs YES) | 434 | ROR | 0.81 | 95%CI | 0.74 | 0.89 | EV < 1 |
| 80 | 72 | SS5 (NO vs YES) | 301 | ROR | 0.82 | 95%CI | 0.76 | 0.9 | EV < 1 |
